# Optimising Multi-stakeholder Practices in Patient Engagement: A Gap Analysis to Enable Focused Evolution of Patient Engagement in the Development and Lifecycle Management of Medicines

**DOI:** 10.1007/s43441-021-00313-9

**Published:** 2021-06-28

**Authors:** S. D. Faulkner, C. A. C. M. Pittens, N. S. Goedhart, E. H. Davies, E. Manning, A. Diaz-Ponce, Maria Jose Vicente Edo, L. Prieto-Remón, L. Husain, K. Huberman, M. Boudes, M. Subramaniam

**Affiliations:** 1 Radcliffe Primary Care Building, Radcliffe Observatory Quarter, Woodstock Rd, Oxford, OX2 6GG UK; 2 Athena Institute for Health and Life Science, Vrije Universiteit Amsterdam, De Boelelaan 1085, 1081 HV Amsterdam, The Netherlands; 3 UCB Pharma, 8010 Arco Corporate Drive, Raleigh, NC 27617 USA; 4 Alzheimer Europe, 14 Rue Dicks, 1417 Luxembourg, Luxembourg; 5 Aragon Health Sciences Institute, Instituto Aragones de Ciencias de La Salud (IACS), Zaragoza, Spain; 6 Aparito, Unit 11-12 Gwenfro, Wrexham Technology Park, Wrexham, LL13 7YP WAL UK; 7 European AIDS Treatment Group, Av des Arts 56-4c, 1000 Brussels, Belgium; 8 European Patients’ Forum, Chaussée d’Etterbeek 180, Brussels, Belgium; 9 Bayer AG. Medical Affairs & Pharmacovigilance, Mullerstrasse 178, 13353 Berlin, Germany

**Keywords:** Patient engagement, Medicine research and development, Medicines lifecycle, Gap analysis, Practices, Processes

## Abstract

**Background:**

The PARADIGM consortium aimed to make patient engagement in the development and lifecycle management of medicines easier and more effective for all, with the development of new tools that fulfil robustly defined gaps where engagement is suboptimal.

**Aims:**

To generate an inventory of gaps in patient engagement practices and process from existing global examples.

**Methods:**

A large set of criteria for effective patient engagement previously defined via a multi-stakeholder Delphi method, were mapped under fourteen overarching themes. A gap analysis was then performed by twenty-seven reviewers against the resulting forty-six mapped criteria, on a sample of seventy initiatives from global databases.

**Results:**

An inventory of gaps was identified including contextual information as to why the gaps exist. Our work identified general patterns where patient engagement was suboptimal—defined as; fragmented reporting and dissemination of patient engagement activities, and the fundamental principles defined in frameworks or guidance being poorly adhered to in actual practice. Specific gaps were identified for sixteen criteria. Additionally, it was also common to observe primary aspects of a process were addressed for a given criteria (i.e. training for roles and responsibilities) but a secondary context element was lacking (i.e. making training material accessible/understandable/meaningful to all participants).

**Conclusion:**

The results show that the evolution towards meaningful and systematic patient engagement is occurring, yet more importantly they provide clear directional insights to help enhance collaborative practices and co-design solutions. This targeted impact to catalyse a needs-oriented health system that integrates patient engagement at its core is essential.

**Supplementary Information:**

The online version contains supplementary material available at 10.1007/s43441-021-00313-9.

## Introduction

There is increasing consensus among stakeholders that patient engagement (PE) in the development and lifecycle management of medicines (further referred to as medicines development) [1] is critical to ensuring timely patient access to more innovative therapeutic solutions, and delivering meaningful healthcare outcomes for patients [1–4]. There are several different definitions on what PE means, here we define PE as; the effective and active collaboration of patients (including patient advocates, patient representatives and/or carers) in the decisions within the medicines lifecycle, along with all other relevant stakeholders when appropriate [4]). Patients can be involved in all phases of medicines development and lifecycle management, from priority setting (i.e. patient relevant outcomes), research design and planning (i.e. protocol design and patient information material), research conduct and operations (i.e. of Health Technology Assessment (HTA) of value), through to the dissemination of end-results (i.e. post approval communications and publications) [3–13]. This has been acknowledged in several European directives and legislation such as; the Clinical Trials Regulation, the Pharmacovigilance Directive, the Paediatric Use Regulation, the Orphan Medicinal Products Regulation and the Advanced Therapies Regulation [14–18].

It is presumed that PE in medicines development can result in outcomes that better meet the needs of patients, since patients provide a uniquely essential type of expertise (often called *experiential knowledge*)—which can bring new perspective to the scientific and medical expertise available from other stakeholders [4, 12, 19] and adds to the legitimacy of research decision-making processes [19].

Despite such progress, patients and their representatives continue to be a largely underutilized resource of expertise in medicines development, and PE is still not fully embedded across the medicines development lifecycle [9, 10, 20]. There are many initiatives emerging to involve and even co-create with patients*.* Many frameworks and guidelines about how to involve patients in research exist [8]. However, the frameworks are seldom put into practice, and less attention has been paid to understanding how to carry forward principles for good PE into practice, and the overall quality and consistency of existing practices is lacking [7]. Utilizing representatives of all stakeholders involved in PE to identify more definitive and highly impactful focus points where PE is currently suboptimal is key to help address these ‘gaps’ in a robust manner.

In previous work, a public–private partnership, PARADIGM, [21] developed a set of criteria that reflects the needs, expectations and preferences of all relevant stakeholders for effective PE in medicines development where PE is suboptimal at several key stages and involved patient populations. These are as follows; i) research priority setting (RPS)—providing opinion or evidence and/or being part of a group that decides what is important to research, ii) clinical trial design (CTD)—designing protocols, discussing patient burden, discussing patient-related outcomes, iii) early dialogues with regulators and HTA bodies (ED)—early discussions between industry, HTA bodies and regulators (and in some contexts with payers) regarding developmental plans for a medicinal product and to ensure they meet the requirements and, iv) potentially vulnerable patients—in the case of PARADIGM these included (but are not limited to) people affected by dementia [22–24], and young people.

The primary aim of this work was to build upon these previous findings and draw upon an exemplary global sample of PE initiatives and perform a gap analysis to better understand how well these pre-defined criteria were being met in practice. A secondary aim was to provide some additional context as to why the identified gaps might exist and provide insight for future efforts that could specifically address some of those deficiencies in an impactful manner. Thus, forging an informed, focused, step-wise improvement approach to the evolution of PE ecosystem apace in real-time.

This work was conducted within the context of the PARADIGM project. PARADIGM is an innovative medicines initiative (IMI) funded project that and falls under the EU’s horizon 2020 framework. It’s mission is to provide a unique framework that enables structured, effective, meaningful, ethical, innovative, and sustainable patient engagement (PE) and demonstrates the ‘return on the engagement’ for all players [21].

## Methods

**Fig. 1:**
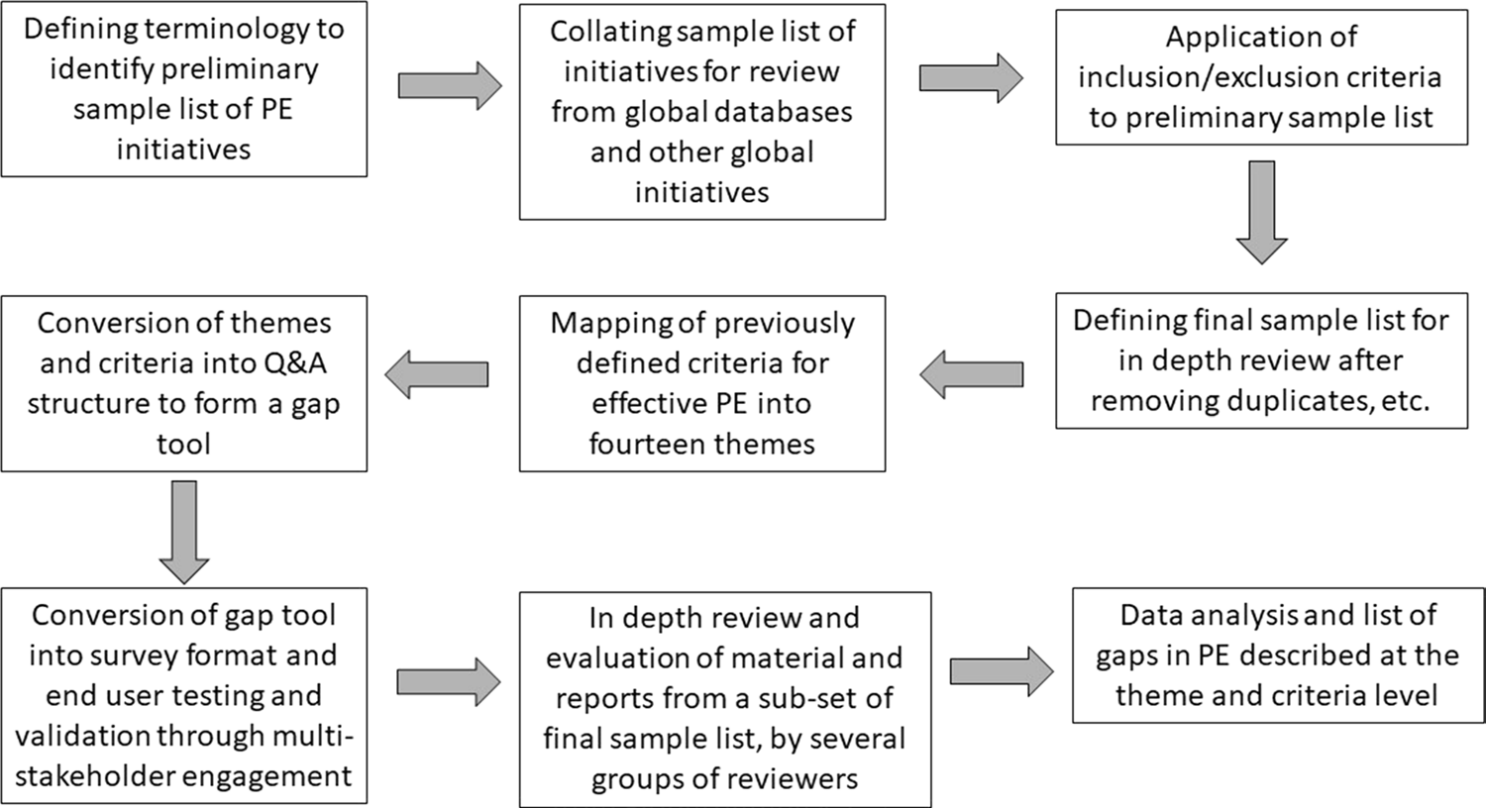
Schematic of three-stage methodology of the gap analysis. 1) Defining the PE initiatives sample group, 2) Gap tool development for evaluation of initiatives, and 3) Data analysis. Abbreviations: PE Patient engagement;

A three-stage approach was followed using qualitative and descriptive quantitative research and analysis methodologies to perform a gap analysis of a relevant and robust sample of PE initiatives in medicines development: 1) Defining the PE initiatives sample group, 2) Gap tool development for evaluation of initiatives, and 3) Data analysis (Fig. 1). Research was undertaken by 27 representatives from the PARADIGM consortium that included patient organisations, the pharmaceutical industry, academia and HTA bodies (https://imi-paradigm.eu/project-partners), between June 2018 and December 2019.

### Stage 1: Defining the PE Initiatives Sample Group

**Fig. 2:**
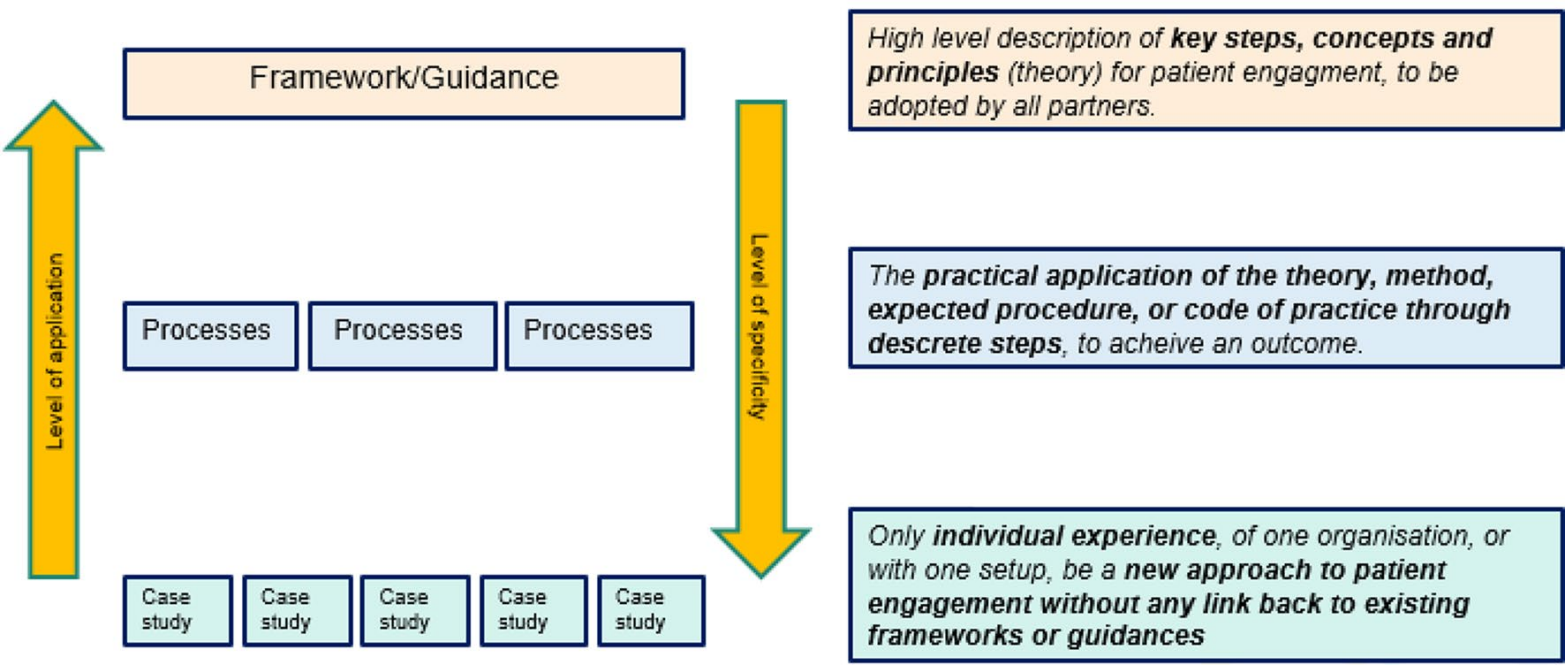
Definitions of practice and process of patient engagement used to refine the primary nature of material reviewed of initiatives included in the gap analysis. These definitions were used to differentiate between practises and processes and general PE material that was for example, advocacy, educational or broadly strategic in nature that did not detail the particular start to finish process of PE activities. See also (21)

A list of agreed criteria for PE had been previously defined by the PARADIGM consortium via a multi-stakeholder, three-stage three panel Delphi method [25]. These criteria are broadly a mix of practice, process and outcomes criteria. Through a multi-stakeholder consultation workshop, it was then agreed to use the terminology of “practices and processes” as presented in Fig. 2 (and from [26]) to broadly define the scope of the initiatives sample group. The identification of PE initiatives occurred in three steps (Table 1 and below) by the first four authors of this article. Initiatives were collated and reviewed where they were categorised to be either a i) framework, ii) guidance, guideline, process, or iii) individual case study (rather than, for example purely advocacy or educational initiatives).

**Table 1:** Inclusion/exclusion criteria applied in three steps to patient engagement initiatives for selection for in-depth review and gap analysis

Step	Inclusion criteria	Exclusion criteria
*1*		
What is the PE initiative type?*	(i) Framework, guidance or guideline (ii) Process (iii) Case study	(i) Training or education** (ii) Advocacy**
Does the PE initiative cover one or more of the three key stages in medicines development?	(i) Research priority setting (ii) Clinical trial design (iii) Early dialogues with regulators and HTA bodies (iv) More than one of these	(i) None of these (ii) Solely benefit/risk (iii) Post market access
Were patients/patient groups/carers directly engaged in the initiative?	(i) Yes to one or more of these	(i) Solely a thought leader (ii) Non-patient expert engagement (iii) Solely being the subject of research
*2*		
What context is the PE occurring in?	(i) Medicines development	(i) Comparative effectiveness research (ii) Other secondary or tertiary care (iii) Health care or health policy
*3*		
(i) Removal of duplicates (ii) Removal of initiatives over 10 years old (iii) Removal of initiatives where insufficient information was available to make a meaningful assessment using the gap tool (i.e. single source, very limited information on the how, why, when and outcomes) and included where information was available but was unable to be shared due to confidentiality issues, or not available within the timeframe of in-depth review)		

Step 1; *or has PE included within it; **If initiatives sole purpose appeared to be training, education or advocacy

*PE* patient engagement, *HTA* Health Technology Assessment

Step 1: An exported list of ~ 300 initiatives were sourced from the two largest existing global databases—Patient Focused Medicines Development (PFMD) [27] and the European Patients Academy (EUPATI) [11]. Further initiatives were identified from the European Federation of Pharmaceutical Industries and Associations (EFPIA) sources (2016 and 2017 Health Collaboration Guides [28, 29]) and from individual organisations or other global initiatives through snowball methods sourced by consortium members.

Step 2: To ensure further focus on initiatives relevant to medicines development that are reflective of current practices and processes, as well as to keep data to a volume that could be analysed in a timely fashion, inclusion/exclusion criteria were applied to the total pool of initiatives identified (Table 1). These two steps resulted in 165 initiatives taken forward for in-depth information gathering beyond what was available in the databases.

Step 3: Further focus removed subsequent duplicates and initiatives where information could not be sufficiently sourced for a meaningful in-depth review (from step 2). This resulted in a final list of 70 initiatives undergoing in-depth review and full analysis.

### Stage 2: Gap tool development for evaluation of initiatives

A gap tool was developed that could be used to provide structured interrogation of the information available for each of the final 70 initiatives. The gap tool was co-created and tested in an iterative manner through two multi-stakeholder workshops and two online webinars with consortium partners, based on foundational conditions and principles for successful collaborations with public and/or patients in decision-making processes [5, 6, 30, 31]. The principles and conditions were used to help map findings from a recent monitoring and evaluation review [2] and output and outcomes criteria from a multi-stakeholder Delphi method into forty-six criteria organised under fourteen themes (Table 2). The details of the original criteria and further context are available in [25]. The final design of the gap tool took the form of a survey with a question and answer structure. A tabulated version of the gap tool is available in supplementary table 1. It consisted of fifteen questions using drop-down answer options that had been adapted from current variables within the PFMD global initiative database [32]. Eleven of these questions were specific to the characteristics of the initiative. Due to the different structure of documents available for in depth review (e.g. framework/guidance, a process/methods document, or a case study) the forty-six assessment questions were structured slightly differently depending on whether the reviewer was assessing material from a i) guidance/framework/process, or ii) case study. Each question and answer was tested for language, readability, consistency and meaningfulness at each stage of development.

**Table 2:** Forty-six criteria for effective patient engagement mapped under fourteen themes that were assessed against seventy patient engagement initiatives using the gap tool

Themes	Criteria within theme
*1. Selection of participant and adequate representation*	(i) Clear description of identification of patient representatives, (ii) engagement of a diverse target population, (iii) Inclusion of views other than patient and, (iv) presence of a description of criteria to identify patient representatives
*2. Empowerment of stakeholders (through availability of training)*	(i) Required competencies, expertise and experiences to perform PE, (ii) training for all stakeholders (including patients) on their roles and responsibilities and, (iii) training material is accessible to all participants
*3. Shared purpose (or aims & objectives)*	(i) Agreement on aims and objectives by all stakeholders (and understandable to all), (ii) aims and objectives focus on patients’ needs and expectations and, (iii) monitoring expectations of aims and objectives
*4. Transparency of roles, scope of involvement and decision-making structure*	(i) Clear definition of roles and responsibilities, ii) clear definition of decision making structures (iii) presence of tools and mechanisms to ensure all understand roles and responsibilities, (iv) explanation and documentation of funding resources, (v) any changes are communicated upfront and, (vi) sharing of outcomes with all stakeholders using appropriate channels and formats
*5. Communication & feedback*	(i) Occurrence of regular communication, (ii) communicating feedback and outcomes in a clear and adapted way, (iii) availability of a named key contact that patients could reach out to, (iv) the opportunity to give regular feedback, (v) availability and communication of legal agreements in a clear and accessible way and, (vi) presence of a co-created dissemination and communication plan for sharing process and outcomes
*6. Feasibility of collaboration and timing of involvement*	(i) Mechanism (e.g. language used, format meeting) to ensure participation of patient representative (ii) Schedule and timelines that respect the need for planning and preparation time (iii) Involvement from start until completion
*7. Sustainability*	(i) Embeddedness of PE in the institution or organization (ii) Allocation of human and financial resources for the long-term continuity (iii) Formation and maintenance of a partnership between all stakeholders
*8. Equal treatment of participants*	(i) Mechanisms in place (e.g. neutral facilitation, open and respectful atmosphere) to ensure a fair deliberative process that allows equal opportunity for all participants’ contribution
*9. Legal & ethical considerations*	(i) Presence of a code of conduct, which clearly states the (ethical) principles (ii) Presence of a privacy policy (iii) Occurrence of procedures to identify and address potential discriminatory, coercive, intimidating, and unethical behavior (iv) Attention for and management of potential conflict of interest, and (v) terms and conditions of all policies and confidentiality agreements are in place
*10. Supportive resources*	(i) Clear, transparent and equitable (fair) financial compensation framework to be in place and made available for patient representatives who participate (ii) Sufficient funding is allocated to governance, administration and relevant operations
**11. Direct outcomes**	(i) Measured outcomes are related to the aims and objectives of the initiative, (ii) reflection of patients’ perspective are clearly defined in the outcomes/result, (iii) outcomes demonstrate a consensus by all participants, and (iv) (mutual) learning on substantive matters is achieved
**12. Impact for medicines development**	(i) Feedback on the implementation of outcomes in practice (ii) Use of metrics to measure impact of PE
**13. Value of PE**	(i) Evidence of value is captured and reported
**14. Learning and reflection**	(i) Methods, tools, and monitoring systems to evaluate PE practice systematically and at appropriate phases of the process (ii) Evaluation outcomes are used to improve future PE practices (iii) Evaluation framework is included and shared among participants (iv) Evaluation criteria are linked to the aims and objectives of the PE practice

Italic = process themes, Bold = outcomes themes

Criteria were mapped from previous work that used a three stage Delphi methodology to define the minimum criteria for effective, meaningful and sustainable patient engagement in medicines development. A detailed description of the original criteria from the Delphi method and additional contextual information can be found at (20). PE = Patient Engagement

Each criteria question followed a basic architecture, “Is there attention to [*criteria*]”. The answer architecture for each question was multiple choice—generally: ‘Yes’, ‘No’, ‘Not able to assess’, or ‘Not relevant to this initiative’. For several questions where there were clearly two elements to the underlying criteria (defined process and nuanced context), an additional answer option was added; ‘Yes, there is attention to [*process element*] but not attention to [*context element*].

All survey questions were compulsory except for themes 12–14 (related to the outcomes of the initiative, (Table 2) that were only answered if metrics or related methods were used within an initiative. In addition to the answer options, open text boxes were available to provide clarifying qualitative information as to why a potential gap existed within a particular initiative. Finally, the gap tool was transposed into an online survey platform (Survey Monkey) for use by the reviewers.

Twenty-seven reviewers from the PARADIGM consortium were randomly allocated a subset of initiatives. They used the gap tool to perform an in-depth review of material based on their cumulative expertise and experience in relevant fields and responsibilities (e.g. pharmaceutical industry, patient organisations, academic, and HTA). Reviewers reviewed initiatives in which their organisation were not involved. In some instances, institutionally available information could not be shared with reviewers due to confidentially restrictions. Where possible, informal conversations were undertaken by the reviewer with the owners of initiatives to gain non-confidential context and clarification. The researchers (first four authors) were available to support the reviewers for any questions they had, e.g. how to interpret available information, what to write down in the open text boxes, whether or not to ask for additional information.

### Stage 3: Data Analysis

Qualitative and descriptive quantitative analysis were performed by the four researchers (first four authors). The descriptive quantitative analysis was differentiated initially for each of the forty-six criteria at the level of either i) a framework, guidance or process, or ii) individual case study.

The output of this analysis was a numerical sum of responses to each answer option for each criteria. For example, the number and percentage of ‘Yes’, ‘No’, etc. responses. A number of the criteria assessments returned a response of, ‘Not able to assess’, rather than binary outputs (i.e. ‘Yes’, or ‘No’ answers). In order to provide further delineation to the findings, a formula was applied to all the numerical responses from each of the forty-six questions. The formula was as follows:

 N (‘Yes’ responses) − N (‘No ‘responses)/N (‘Not able to assess’ responses).

The resulting numerical range of values was determined for all forty-six questions at the level of either i) total number of initiatives (combined framework/guidance/process or case study) or ii) differentiated by, i) frameworks/guidance, processes, or ii) case study. This range was delimited by applying a < 50th or > 50th percentile range. Values below the < 50th percentile range equated to ‘a gap’, while those values above the > 50th percentile equated to ‘no gap’. Second, open text responses were analysed qualitatively for each of the fourteen themes separately. An inductive coding strategy [33] was used to analyse whether the open text responses were supportive or in contrast with the quantitative descriptive analyses. Any marginal or ambiguous qualitative responses were also discussed within the multi-stakeholder review groups for clarity.

Finally, both qualitative and descriptive quantitative findings were integrated and confirmed to be largely consistent throughout. Where a gap occurred at framework/guidance/process, an individual case study, or both, and confirmed by appropriate qualitative findings it was included as a gap.

## Results

The *characteristics* of the initiatives are listed below, followed by the *general* and *contextual gaps* that emerged across the initiatives, and then the more detailed descriptions of the *specific gaps* by theme that were identified.

### Characteristics of Initiatives

**Fig. 3:**
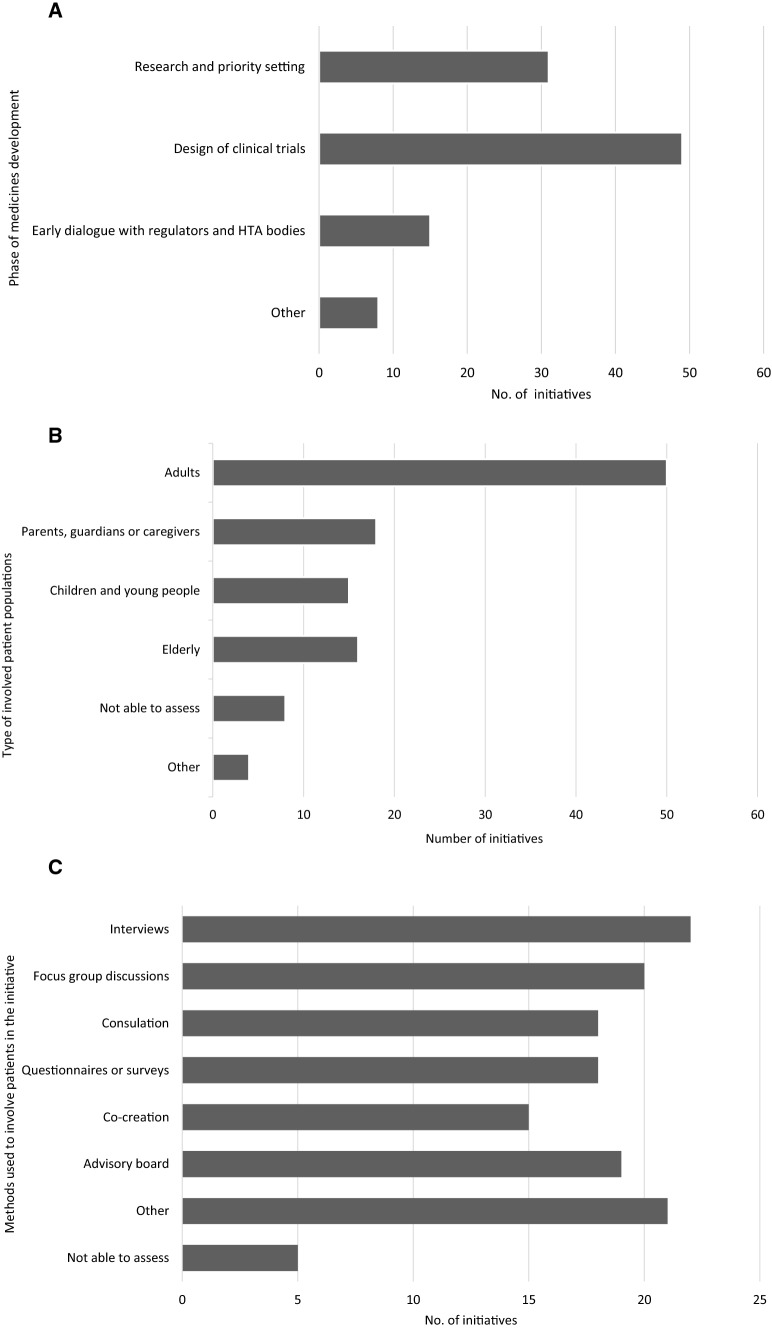
Sample characteristics of the seventy initiates interrogated for gap analysis. Some questions were multiple choice, therefore more than one answer was possible for some questions. **A** Phases of medicine development that the initiative covered. ‘Other’ = relevant to more than one phase. **B** Type of involved patient populations in the initiatives. **C** Methods used or proposed to be used to involve patients in the initiative. Full table summarising all eleven initiative characteristics are in Supplementary Table 2

The full set characteristics are available in supplementary Table 2. Briefly, forty initiatives (57%) were considered individual case studies, eighteen (26%) a framework or guidance and twelve (17%) a process. Overall, initiatives covered at least one key stage in medicines development, either; RPS (30%), CTD (48%), or ED (15%) (Fig. 3A–C). Further examples included were relevant across the whole medicines development lifecycle. Initiatives involved a variety of patient populations (such as individual patients, patient organisations, patient experts), including relevant potentially vulnerable patients (such as elderly, young people, and carers) and diverse disease areas (such as oncology, neurodegenerative, immunology, autoimmune and vision related).

### General Gaps

Two important and consistent general patterns emerged, supported by open text responses and reviewer feedback that were not associated with a specific criterion.

Firstly, there is a general lack of detailed reporting and dissemination of information about PE activities. For example, regarding the context of the engagement, details on the process for communication and decision making, methods used to ensure diversity of the involved patients and stakeholders, and the outcomes and learnings of the activity. Information on these elements was either not available or discoverability in the public domain was difficult. When this information existed, it was often unstructured, spread across a variety of information sources (webpage, cases reports, templates, and summary minutes) or lacked the necessary details to make it possible for others to fully understand the key elements of the PE activity. Qualitative responses helped explain the gap. Some responses suggested that there was a lack of granularity in the reporting that was done on the PE process and activity itself—especially when reporting in the public domain, or that a given practice/process had occurred to some extent but was not formally documented due to resource constraints.

Secondly, there was also a general lack of evidence that principles defined in overarching frameworks/guidance/guidelines were implemented in individual case studies. Only a few examples included the provision of additional resources (e.g. a reference list of documents) or links to other platforms or initiatives on how principles defined within a guidance or framework might be actioned in practice. With respect to case studies, there was little or no referencing back to existing guidelines or frameworks to indicate that they had been followed. Qualitative responses helped partly explain this gap, capturing that often the processes or related guidance were under development but were not yet implemented or systemically operationalised.

### Context-Specific Gaps

Most of the criteria assessed were singular in nature and only five of the forty-six criteria contained two elements—a defined process element and a nuanced context element. For example, if the primary process element of a criteria was *training for their roles and responsibilities***,** the secondary context element was *training material is accessible to all participants.* Overall, initiatives often reported the process element, but not the context element. Responses confirmed that attention to the context element, for example, information about any *specific consideration to patient inclusivity and accessibility* was often limited meaning the criteria was not fully met.

Finally, with respect to the three stages of medicines development discussed herein, despite a higher number of initiatives that covered CTD compared to RPS or ED, the general ratios of answers—‘Yes’, ‘No’ or ‘Not able to assess’—remained the same for each criterion assessed. Consequently, no obvious differences emerged towards one key stage over another.

### Criteria-Specific Gaps

Sixteen gaps were identified from the forty-six criteria (~ 40% of total). The gaps spread across nine of the fourteen overarching themes. In the other five themes generally criteria had been addressed in the majority of the initiatives analysed—hence no definitive gap was confirmed*.* Where gaps were identified in some cases this was at the level of both frameworks/guidance/processes and case studies. However, a majority of gaps were confirmed to occur at the level of case studies. A description of these gaps at the theme level, accompanied by some illustrative qualitative findings is detailed below and in Table 3.

**Table 3:** Identified gaps in patient engagement practises and process with example qualitative quotes reported at the theme level to support the identified gaps

Category (theme)	Criteria with gap identified (was there attention to…..?)	Illustrative open text responses supporting identified gap
Selection of participants and adequate representation	Clear description of the criteria followed to identify patient representatives needed	“Unlikely diversity [is] reflected as only patient advocates involved. Detail about who and how they were selected [is] not available”
Empowerment of stakeholders	Training for their roles and responsibilities with training material accessible to all participants*	“ ….assumed patient organisations train their patient representatives”
Transparency of roles, scope of involvement, and decision-making structure	Communicating any changes that could occur during the PE initiative up-front	“owner of the initiative informed that the information is described in confidential material that cannot be shared”**
Communication and feedback	Legal agreements written in a clear and accessible way and adapted to the target population	“legal agreements existed and included details but I can’t assess if they were suited to the needs of participants.”
	Include a dissemination and communication plan sharing the process and outcomes	“Process and outcomes are shared but detail about plans for this are not available “
Sustainability	Ensure the formation and maintenance of a partnership between all stakeholders	“It was sponsored by a company with a specific goal and for a limited time period” and “limited to the funding from the EU program” “This is part of the company sponsorship with organisation[x], so although this particular activity was a one off, it is part of a broader relationship between organisation[x] and the company”
Legal and ethical considerations	Code of conduct, which clearly states the (ethical) principles, governance requirement, rules and procedure of participation for all stakeholders involved	“To my knowledge specific forms, guidelines and templates were not created. However, given the organisations had official collaborative relationships as Working Party members, it is understood that legal and privacy contracts would have been managed as appropriate for any such arrangement”
	Privacy policy that describes policy to maintain data privacy of engaged patients in the engagement	“Contracts were signed between the patient organisation and company. However, legal agreements are not accessible to patients/patient organisations”
	Identification and addressing potential discriminatory, coercive, intimidating, and unethical behaviours, towards all stakeholders, before, during and after their participation?	
	Management of potential conflicts of interest (up to avoidance)? disclosure, transparency and accountability	
	Presentation of the terms and conditions of all policies and confidentiality agreements, in a clear and accessible way to the stakeholders involved	
Supportive resources	Clear, transparent and equitable (fair) financial compensation framework to be in place and made available for patient representatives who participate	“Three patients in the steering committee received financial compensation, but compensation framework is not available in the information collected.”
Impact for R&D	Propose metrics to measure impact of PE	“Company [x] has created an evaluation survey to collect information on learnings and outcomes from a sponsor and member perspective, self-reported results are available. The survey doesn't measure specific metrics, but does include some questions that are proxies for impact.”
Learning and reflection [on the PE practices]	Propose methods, tools and monitoring systems to evaluate the PE practice systematically and at appropriate phases of the process	“They state a survey was used to get feedback from stakeholders about how to improve the design of the initiative and so on, but a framework for evaluation is not discussed” “Evaluation was informally processed by feedback and sharing among the leads and learnings are being incorporated into the 5 year plan” and, “They evaluated through live discussion as they progressed; no official criteria or assessment”
	Propose an evaluation framework for evaluation of the PE initiative	
	Link between the evaluation criteria and the aims and practices of the PE practice	

Gaps are identified and arranged under their respective overarching theme. Example qualitative responses from open text reported at the theme level are included and in some cases are relevant to more than one gap within a theme

*Taking into consideration languages, impairments, literacy levels, cultural background and the circumstances of (vulnerable) patients involved

**Open text responses reported at the theme level only. Semi-quantitative analysis revealed the gap at all levels

#### Selection of Participants and Adequate Representation

At the theme level, for both frameworks/guidance/processes and individual case studies, documented information on who was involved, how they were identified, and how the diversity of views and experiences of participants was ensured, was both sporadic and often incomplete. Specifically, a gap emerged for the criterion *clear description of identification of patient representatives* at the level of case studies. It was reported that some stakeholders, such as regulators, have clear procedures/protocols for patient organisation identification, which are available publicly. While for other stakeholders, for example the pharmaceutical industry, patient identification and selection can be outsourced to third parties along with other PE-related activities. This could result in some loss of clarity and control over protocol implementation. For example, some responses suggested that only patient representatives had been involved, rather than individual patients. Additionally some responses suggested that there was only attention to diversity of the condition, gender and race, rather than other factors of diversity, such as including vulnerable populations. As one response paraphrased (for the initiative assessed), “[It is] unlikely diversity reflected as only patient advocates involved. Detail about who and how they were selected not available” (Table 3).

#### Empowerment of Stakeholders

Overall the analysed frameworks/guidance/processes, paid more attention to the three criteria under this theme than in the case studies, (Table 2). However, gaps were still identified for two of the three criteria at the level of case studies: *training for their roles and responsibilities* and *training material is accessible to all participants*. With respect to training, some responses revealed that the occurrence of actual training for roles and responsibilities was relatively low. For example, some responses reported assumptions that patient organisations train their own patient representatives. In some other instances, it was reported that training was not needed for the activity, or that the engaged patients had been selected based on already having the skills or knowledge required. With respect to material being accessible, it was consistently difficult to confirm whether training materials were accessible to all participants,

#### Transparency of Roles, Scope of Involvement, and Decision-Making Structure

At the theme level there was a general lack of information documented or made available for the five criteria here. However, a gap was identified for the criterion *communicating any changes that could occur during the PE initiative up-front* with respects to frameworks/guidance/processes and case studies. For example, one response suggested that the topic of communicating changes was not overt in public information, while another response suggested that, “the owner of the initiative informed that the information is described in confidential material that cannot be shared”. Interestingly, at the theme level it was also reported qualitatively that patients were not always involved in decision-making, and at times it was deemed not relevant to do so—as supported by the following qualitative data: “There was no follow-up communication or involvement in any decision-making”. However, quantitatively this was not found as a gap.

#### Communication and Feedback

Most of the analysed initiatives addressed the criterion, *occurrence of regular communication*. There were gaps, however, for two of the six criteria specifically. For the criterion, *availability and communication of legal agreements in a clear and accessible way*, confirmatory information was often sporadic or unavailable. The qualitative responses mentioned assumptions that legal and ethical considerations were in place based on the longstanding relationship between the engaging stakeholder and patients and their representatives: *“*To my knowledge specific forms, guidelines and templates were not created. However, given the organisations had official collaborative relationships [name of organisation], it is understood that legal and privacy contracts would have been managed as appropriate for any such arrangement*”.* In addition, there was often ambiguity as to whether those legal agreements were understandable and accessible for the patients involved. For the criterion, *presence of a co-created dissemination and communication plan for sharing process and outcomes,* it was reported that feedback (to patients) on process and outcomes did not always happen;—remaining internal to the engaging organisation. Where feedback was shared, it was often unclear if dissemination and communication plans were used or available to patients: *“*Process and outcomes are shared but detail about plans for this are not available* “* (Table 3).

#### Sustainability

At the theme level, most PE initiatives appear to have some form of grounding (people, resources, or strategy) in the organisation. However, a gap was identified for the criterion, *formation and maintenance of a partnership between all stakeholders.* It was reported that case studies were often one-off events and were not always designed to be part of a sustainable partnership between the engaging stakeholder and the patient organisation/patient. Reasons given for this gap included limited funding and changing strategy or disease focus. Encouragingly, some responses suggested that organisations are in the process of building longer term bilateral partnerships that include more established foundations, materials, and resources.

#### Legal and Ethical Considerations

At the theme level, gaps were identified across all five criteria assessed.

There was a general lack of reporting of or available information to confirm attention to legal and ethical procedures: code of conduct, privacy policy, procedures to identify and address potential discriminatory, coercive, intimidating, and unethical behaviour, management of potential conflict of interest, and terms and conditions of all policies and confidentiality agreements are in place.

Similarly to theme 4, it was often reported that it was assumed by the reviewer that all legal and ethical considerations were in place, but often this assumption could not be confirmed by the owner of the initiative. A supportive quote exemplifies this, “To my knowledge specific forms, guidelines and templates were not created. However, given the organisations had official collaborative relationships as Working Party members, it is understood that legal and privacy contracts would have been managed as appropriate for any such arrangement”. It was also mentioned several times that legal and ethical documentation was in place, but only limited details were given as to the specific types of documents. For example, “Contracts were signed between the patient organisation and company. However, legal agreements are not accessible to patients/patient organisations”. Finally, it was highlighted that where information on these technical documents was available it was not possible to assess whether this was actually written in easy and accessible language for patients.

#### Supportive Resources

At the theme level qualitative responses suggested that that generally participants received compensation for the PE activity. However, a gap was identified for the criterion of *whether there was attention to a clear, transparent and equitable (fair) financial compensation framework***.** A majority of framework/guidance/process lacked specific details of implementing compensation mechanisms. It became apparent that this is difficult because the context of each initiative has different needs with regard for financial compensation. However, it was reported that adherence to any existing or published financial compensation framework was ambiguous.

#### Impact on Medicines Development

At the theme level not all initiatives reviewed were relevant as not all considered or used mechanisms to measure impact on medicines development. For those relevant initiatives where some consideration was mentioned, there was generally attention to *feedback on the implementation of outcomes.* However, the *use of metrics to measure impact of patient engagement* seemed to be much more challenging and revealed a clear gap*.* In the frameworks/guidance/processes analysed there was very limited attention to the use of metrics; only two frameworks were mentioned. Elsewhere in case studies metrics were largely absent and, where reported, were sporadic, incomplete or singular outcomes were used for proxies of impact.

#### Learning and Reflection on the Patient Engagement Practices

At the theme level there were gaps in three of the four criteria across both frameworks/guidance/processes and case studies—(i) *propose methods, tools and monitoring systems to evaluate the patient engagement practice*, (ii) *propose an evaluation framework*, and (iii) *link between the evaluation criteria and the aims and practices of the patient engagement practice.* It was reported that mechanisms exist and are used to evaluate PE activities, such as surveys. However, it was often unclear or unreported if this was part of a larger (formal) evaluation framework. Less formal feedback mechanisms were often used by the engaging organisation but no official evaluation criteria were mentioned. For example, *“*Evaluation was informally processed by feedback and sharing among the leads and learnings are being incorporated into the 5 year plan*”*, and, *“*They evaluated through live discussion as they progressed; no official criteria or assessment [was used]*”* (Table 2).

## Discussion

The value of this global project is several fold. First, we elevated the insight as to where PE is suboptimal beyond a single-consensus method (for example one workshop or survey involving one or two stakeholder groups). We utilised a longitudinal methodology and the power of a diverse and informed public–private partnership to combine previously defined criteria for effective PE with a detailed gap analysis of a large, global sample of PE initiatives. Second, the process of the gap tool development and in depth review in itself has been a valuable driver of meaningful dialogue and knowledge gain of the current PE landscape for all of the involved multi-stakeholder organisations. This work has revealed that a clear shift is taking place towards more PE in medicines development, highlighting less recognised gaps, nuanced by both process and context factors (see Table 3). Third, the gap tool in itself is a worthy standalone outcome of the analysis. In line with the conclusions of Greenhalgh et al. [8], this analysis utilised available evidence on criteria and frameworks for successful PE and co-designed our own framework which fit the purpose of our study, and had broad support from the stakeholders in the consortium. The resulting identified gaps (and the criteria they are based upon) can be used as a type of organisational ‘sense check’ or ‘check list’ in combination with existing tools and frameworks [34, 35] to help organisations identify improvements in the way some elements of PE is currently being conducted or shape PE practices beforehand. Taken together, the outcomes provide a clear stimulus and enabler to target and fulfill gaps in PE pragmatically and synergistically as part of a broader strategy, involving all stakeholders groups, to improve PE in ‘real time’ [7, 20].

### Reflection on Identified Gaps

The identified list of gaps are presented in Table 3. Several identified gaps stand out and will require particular attention and coordinated action by any future efforts for improvement: (i) less attention to contextual elements, (ii) Inconsistent reporting and dissemination of PE activities, and (iii) monitoring and evaluation of PE.

### Less Attention to Contextual Elements

Where criteria contained both a process element and a secondary contextual element, while the process element was generally adhered to it was often this contextual element that appeared to be poorly adhered to or missing. For example, evidence was generally missing or unavailable regarding attention to; inclusion of all relevant patient populations (e.g. potentially vulnerable patients, and carers), addressing the specific needs and circumstances (including language, timing, format, etc.), or other considerations of those patients (i.e. that patients had access to all relevant information in accessible formats and in a timely manner). This demonstrates that already much consideration is in place to a broad range of relevant PE criteria but that inclusion and the provision of adequate support require special attention and additional efforts to further meaningful PE. Disease and population specific umbrella organisations [36–39], address some of these pressing issues around the inclusion of vulnerable populations [22, 23] through advocacy, training/provision of information and support and tool development. Yet much more is still needed across the board. These include improved education, dissemination and knowledge sharing of the needs of these populations; tools and guidelines to help organisations to identify and engage with populations in an appropriate and respectful manner; and training for all stakeholders to ensure the associated capacities and capabilities are in place to do so consistently. References to diversity and representation should not be understood as being just about the number of people who should be involved, but rather about whether the range of experiences and perspectives from a wide range of people have been covered (e.g. people from different socio-economic backgrounds, level of educations, etc.). This is relevant between and within patient groups, including when conducting PE with vulnerable populations. The challenge with training is to ensure that it is empowering and does not, rather than empower, discourage patients from getting involved or discriminate against certain groups [24].

#### Inconsistent Reporting and Dissemination of PE Activities

The finding of inconsistent reporting and dissemination of PE activities in the public domain and the general lack of discoverability of information speaks to a much broader set of issues across many of the identified gaps and PE as a whole. These centre on improved communication about every aspect and every step of the PE activity itself – most relevant to individual cases studies—so that the process and outcomes of the PE activity are communicated in a timely and appropriate manner to all involved. It also includes missed opportunities for broader knowledge gain and continuity within and across organisations, where for example, practises or processes were reported to occur but were not formally documented, or knowledge lost with changes in personnel. Furthermore gaps were found more consistently at the level of case studies than frameworks/guidance/process, and with many supportive quotes, suggests that published principles for PE may not necessarily be used in practise. The reporting and dissemination in the public domain of all PE activities, that are easily discoverable is essential for open and shared learning, and thereby driving the continued learning and improvement culture of PE and embedding of accepted principles. Given the broad reach and diversity of stakeholders involved in steering the PE ecosystem, the responsibility lies with collaborative, diverse and inclusive multi-stakeholder leadership [40] to more proactive knowledge sharing in the public domain (for example, EUPATI [11] and PFMD [32]).

#### Monitoring and Evaluation of PE

The most prominent identified gaps under themes of *impact for medicines development* and *learning and reflection* are highly relevant to the development of a monitoring and evaluation framework for PE. There was a paucity of defined practices for learning and reflection reported here. Conclusively, there is room for improvement to determine effective metrics that clearly link any evaluation criteria with the primary aims of the patient engagement practice and that these are used as part of learning practice. A few initiatives are visible regarding the development of frameworks and other PE quality guidance’s [41, 42]. However, there are notably limited frameworks and metrics to demonstrate quantitative [1] and qualitative [2, 8] value measures of PE. Caution is needed here that the focus should be on learning and improving and not only demonstrating numerical value.

#### Methodological Considerations

There are several limitations to the scope of this work. Initiatives were sourced from existing databases and contemporary examples provided by some organisations. These were dominated by Western EU and US examples and lack those from other regions of the EU such as Central Eastern European (CEE), Asia, or material not presented in English. Due to the large number of criteria being assessed against, a pragmatic balance of the breadth of initiatives to asses and the depth of analysis of each initiative was prioritised. Initiatives were limited to PE in medicines development and those involved in the key stages of RPS, CTD or ED. Initiatives covering other areas of PE such as comparative effectiveness research (CER) and patient preference were excluded as they are addressed elsewhere [43, 44]. The limited number of initiatives from some disease areas, potentially vulnerable populations and those with relevance to ED did not allow additional differentiation of gaps specifically at these levels.

The quality and structure of the material available for in-depth review varied considerably. There were numerous instances where comprehensive material was not available. It is acknowledged that this biases the analysis as the full picture may not be represented. Performing secondary in-depth interviews with the owner(s) of a subset of initiatives where clear gaps had been identified could have further enriched our understanding of the context and broader barriers to PE. Unfortunately, strict time limits and changes in institutional contacts prevented this further work in several instances.

Due to the large number of responses to some criteria being, ‘not able to assess’, additional delineation was given to the descriptive quantitative analysis through applying a formula to raw response numbers. Understandably, this secondary approach artificially constrains and biases the data towards identifying a gap than not, and other methods may have resulted in different conclusions. The descriptive quantitative data was, however, analysed separately from the qualitative data, by different analysts, and when subsequently cross-checked confirmatory conclusions were reached.

## Future Implications

The PARADIGM consortium has incorporated some of these gaps into subsequent efforts for co-creation of new tools that include; managing conflicts of interest, codes of conduct, identification of the right patient match for the right patient engagement activity, reporting and dissemination, lay summaries of legal agreements, and a monitoring and evaluation framework. This way, a constant learning and improvement culture is sustainable in which gaps will be addressed effectively, tools can be fit for purpose, and PE will be meaningful and impactful for those who leverage the knowledge.

## Conclusion

The list of gaps here show the desired evolution in PE is occurring—more than half of the criteria assessed against appeared to be adhered to for at least some or most of the time. Yet more importantly the identified gaps provide clear directional insights to enhance collaborative practices and co-design solutions for targeted impact that will further catalyse a needs-oriented health system, where PE plays a key role [34, 35, 43, 45–47].

## Supplementary Information

Below is the link to the electronic supplementary material.

Supplementary file1 (DOCX 75 kb)
