# Supplementary material for: Optimising Multi-stakeholder Practices in Patient Engagement: A Gap Analysis to Enable Focused Evolution of Patient Engagement in the Development and Lifecycle Management of Medicines
Source: Ther Innov Regul Sci. 2021 Jun 28;55(6):1165–79. doi: 10.1007/s43441-021-00313-9 (PMC8492561; doi:10.1007/s43441-021-00313-9)
Supplement: Supplementary file 1 — Supplementary file1 (DOCX 75 kb) [file 43441_2021_313_MOESM1_ESM.docx]

**TIRS**

**Title**: Optimising multi-stakeholder practices in patient engagement: A gap analysis to enable focused evolution of patient engagement in the development and lifecycle management of medicines

**Authors** Faulkner SD^1*^ (PhD) Pittens CACM^2^(PhD) Goedhart NS^2^, Davies EH^1,6^, Manning E^3^, Diaz-Ponce A^4^, Vicente-Edo Maria J^5^, Prieto-Remón L^5^, Husain L^1^, Huberman K^7^, Boudes M^8^ and Subramaniam M (MPharm)^9^

**Institutional affiliations**:

^1^Radcliffe Primary Care Building, Radcliffe Observatory Quarter, Woodstock Rd, Oxford OX2 6GG, UK +44 (0)1865 617825* Corresponding author

^2^Athena Institute for Health and Life Science, Vrije Universiteit Amsterdam, De Boelelaan 1085, 1081 HV, Amsterdam, the Netherlands

^3^UCB Pharma, 8010 Arco Corporate Drive, Raleigh, NC, 27617, USA

^4^Alzheimer Europe, 14 Rue Dicks, L1417, Luxembourg

^5^ Aragon Health Sciences Institute, Instituto Aragones de Ciencias de la Salud (IACS)

^6^Aparito, Unit 11-12 Gwenfro, Wrexham Technology Park, Wrexham, Wales LL13 7YP

^7^European AIDS Treatment Group, Av des Arts 56-4c, 1000 Brussels, Belgium

^8^European Patients’ Forum, Chaussée d’Etterbeek 180, Brussels, Belgium

^9^Bayer AG. Medical Affairs & Pharmacovigilance, Mullerstrasse 178, 13353 Berlin, Germany

**(4) the name and address, including email address, of the author to whom correspondence is to be sent;**

^1^Radcliffe Primary Care Building, Radcliffe Observatory Quarter, Woodstock Rd, Oxford OX2 6GG, UK +44 (0)1865 617825* Corresponding author

|  |  | Sub-theme | New proposal | Questions process/guidance’s | Answer format process/guidance’s | Questions Case studies | Answer format Case studies |
| --- | --- | --- | --- | --- | --- | --- | --- |
|  |  |  |  |  |  |  |  |
|  | **Stakeholder Representation**  **Process criteria** | Selection of participant and Adequate representation | - A clear description of the **criteria followed to identify the needed patient representatives**. - The patient representatives engaged reflect the relevant **diversity of the target population** (included but not limited to cultural background, socio-economic status, gender, age, ethnicity, educational level, disease, disability, vulnerability, etc.) and their range of perspectives. - If relevant, **views of others than patients** (e.g. carers, parents, etc.) are also considered - A clear description of the **criteria followed to identify patient representatives** is provided | 1. Is there an attention for including relevant diversity of the target population (included but not limited to cultural background, socio-economic status, gender, age, ethnicity, educational level, disease, disability, vulnerability, etc.) and their range of perspectives? 2. Is there attention to include views of others than patients (e.g. carers, parents, etc.)? 3. Is there attention for including a clear description of criteria to identify patient representatives needed? | 1a Yes, there is attention for relevant diversity of population.  1b No, there is no attention for relevant diversity of population.  1c It is not possible to assess based on the available information.  1d This question is not relevant to this initiative.  2a Yes, there is attention for the views of others.  2b No, there is no attention for others’ views.  2c It is not possible to assess based on the available information.  2d This question is not relevant to this initiative.  3a Yes, there is attention for criteria to identify patients.  3b No, there is no attention for criteria to identify patients  3c It is not possible to assess based on the available information.  3d This question is not relevant to this initiative. | 1. The included patients reflect the relevant diversity of the target population (included but not limited to cultural background, socio-economic status, gender, age, ethnicity, educational level, disease, disability, vulnerability, etc.) and their range of perspectives? 2. If relevant, did the initiative consider views of others than patients (e.g. carers, parents, etc.)? 3. Did the initiative include a clear description of the criteria followed to identify patient representatives needed | 1a Yes relevant diversity of population was reflected.  1b No, relevant diversity of population was not reflected.  1c It is not possible to assess based on the available information.  1d This question is not relevant to this initiative.  2a Yes, the views of others were considered.  2b No, there was no consideration of others’ views.  2c It is not possible to assess based on the available information.  2d This question is not relevant to this initiative.  3a Yes, a clear description of criteria for identifying patients was included.  3b No criteria for identifying patients were included.  3c It is not possible to assess based on the available information.  3d This question is not relevant to this initiative. |
|  |  |  |  | **FREE TEXT OPTION (MAX 250 WORDS)**  If the answer was “NO” or “Not possible to assess” or if the question is “Not considered relevant to that specific initiative” to the previous questions please provide additional specific information. Examples include: limited detail in the report, lack of clarity, or ambiguity of the ‘who’, ’what’, ’when’ and ‘how’.  Please also describe what other “gaps” or aspects of the gap need to be considered.  -Why information on criteria ‘X’ was not available?  -Why criteria ‘X’ or ‘Y’ was not undertaken, considered, or followed? | | **FREE TEXT OPTION (MAX 250 WORDS)**  If the answer was “NO” or “Not possible to assess” or if the question is “Not considered relevant to that specific initiative” to the previous questions please provide additional specific information. Examples include: limited detail in the report, lack of clarity, or ambiguity of the ‘who’, ’what’, ’when’ and ‘how’.  Please also describe what other “gaps” or aspects of the gap need to be considered.  -Why information on criteria ‘X’ was not available?  -Why criteria ‘X’ or ‘Y’ was not undertaken, considered, or followed? | |
|  |  | Empowerment of stakeholders (through availability of training) | - The **competencies, expertise and experiences** required to perform patient engagement by all participants is defined   All **stakeholders - including but not limited to patients are** offered / undertake **training** for their roles and responsibilities as required   - The **training material used is adapted**, comprehensible and accessible to all participants taking into consideration impairments, language, literacy levels, cultural background and the circumstances of (vulnerable) patients involved | 1. Is there attention for identifying the relevant competencies, expertise and experiences that are required to perform patient engagement by all participants? 2. Is there attention for including training for all participants for their roles and responsibilities with training material accessible to all participants taking into consideration of languages, impairments, literacy levels, cultural background and the circumstances of (vulnerable) patients involved? | 4a Yes, there is attention for identifying competencies, expertise and experiences.  4b No, there is no attention for identifying competencies, expertise and experiences.  4c It is not possible to assess based on the available information.  4d This question is not relevant to this initiative.  5a Yes, there is attention for including training opportunities for all participants and in appropriate formats.  5b Yes, there is attention for including training opportunities for all participants, but there is no attention for accessible or appropriate formats.  5c No, there is no attention for including training opportunities.  5d It is not possible to assess based on the available information.  5e This question is not relevant to this initiative. | 1. Did the initiative identify the relevant competencies, expertise and experiences that are required to perform patient engagement by all participants? 2. Did the initiative include / offer training for all participants for their roles and responsibilities with training material accessible to all participants taking into consideration of languages, impairments, literacy levels, cultural background and the circumstances of (vulnerable) patients involved? | 4a Yes, all required competencies, expertise and experiences were identified.  4b No, competencies, expertise and experiences were not identified.  4c It is not possible to assess based on the available information.  4d This question is not relevant to this initiative.  5a Yes, training opportunities were made available for all participants and in appropriate formats.  5b Yes, training opportunities were made available for all participants, but did not consider accessible or appropriate formats.  5c No training opportunities were made available.  5d It is not possible to assess based on the available information.  5e This question is not relevant to this initiative. |
|  |  |  |  | **FREE TEXT OPTION (MAX 250 WORDS)**  If the answer was “NO” or “Not possible to assess” or if the question is “Not considered relevant to that specific initiative” to the previous questions please provide additional specific information. Examples include: limited detail in the report, lack of clarity, or ambiguity of the ‘who’, ’what’, ’when’ and ‘how’.  Please also describe what other “gaps” or aspects of the gap need to be considered.  -Why information on criteria ‘X’ was not available?  -Why criteria ‘X’ or ‘Y’ was not undertaken, considered, or followed? | | **FREE TEXT OPTION (MAX 250 WORDS)**  If the answer was “NO” or “Not possible to assess” or if the question is “Not considered relevant to that specific initiative” to the previous questions please provide additional specific information. Examples include: limited detail in the report, lack of clarity, or ambiguity of the ‘who’, ’what’, ’when’ and ‘how’.  Please also describe what other “gaps” or aspects of the gap need to be considered.  -Why information on criteria ‘X’ was not available?  -Why criteria ‘X’ or ‘Y’ was not undertaken, considered, or followed? | |
|  | **Process structure** | Shared purpose (or aims & objectives) | **Agreement on the aims and objectives** by all stakeholders is in place **and understandable** to all stakeholders involved.   - The **aims and objectives** focus on patients’ needs and interests and **meet patients’ expectations.** - **Monitoring that the aims and objectives are met and meet the expectations** of all stakeholders in in place (feedback loop) | 1. Is there attention for agreeing on aims and objectives in consultation by all stakeholders collectively? 2. Is there attention for monitoring the expectations of all in regard to meeting the aims and objectives? | 6a Yes, there is attention for agreeing on aims and objectives in consultation by all stakeholders collectively.  6b No, there is no attention for agreeing on aims and objectives.  6c It is not possible to assess based on the available information.  6d This question is not relevant to this initiative.  7a Yes, there is attention for monitoring expectations of all stakeholders in regard to meeting the aims and objectives.  7b No, there is no attention for monitoring expectations of all stakeholders in regard to meeting the aims and objectives.  7c It is not possible to assess based on the available information.  7d This question is not relevant to this initiative. | 1. Were the aims and objectives agreed upon in consultation by all stakeholders collectively? 2. The expectations of all stakeholders were monitored in regard to meeting the aims and objectives? | 6a Yes, aims and objectives were agreed upon in consultation by all stakeholders collectively.  6b No aims and objectives were agreed upon.  6c It is not possible to assess based on the available information.  6d This question is not relevant to this initiative.  7a The expectations of all stakeholders were monitored in regard to meeting the aims and objectives.  7b No expectations of all stakeholders were monitored in regard to meeting the aims and objectives.  7c It is not possible to assess based on the available information.  7d This question is not relevant to this initiative. |
|  |  |  |  | **FREE TEXT OPTION (MAX 250 WORDS)**  If the answer was “NO” or “Not possible to assess” please provide additional specific information. Examples include: limited availability of the report, lack of clarity, and ambiguity of the ‘who’, ’what’, ’when’ and ‘how’.  Please also describe what other “gaps” or aspects of the gap need to be considered.  -Why information on criteria ‘X’ was not available?  -Why criteria ‘X’ or ‘Y’ was not undertaken, considered, or followed? | | **FREE TEXT OPTION (MAX 250 WORDS)**  If the answer was “NO” or “Not possible to assess” please provide additional specific information. Examples include: limited detail in the report, lack of clarity, or ambiguity of the ‘who’, ’what’, ’when’ and ‘how’.  Please also describe what other “gaps” or aspects of the gap need to be considered.  -Why information on criteria ‘X’ was not available?  -Why criteria ‘X’ or ‘Y’ was not undertaken, considered, or followed? | |
|  |  | Transparency of roles, scope of involvement and decision-making structure | - R**oles and responsibilities are clearly defined for all** - **Specific tools** (e.g. roles and responsibility charts, procedural guidance documents, protocols) **and mechanisms** (e.g. meetings, workshops, training sessions) to ensure that all participants **understand their own and others’ roles and responsibilities.** - **Funding resources** are clearly **documented and explained** to all stakeholders involved prior to involvement, and any **changes that occur during the practice are communicated** up-front - The o**utcomes are shared** with all the stakeholders involved using appropriate channels and formats suited to patient perspectives’ circumstances and needs. | 1. Is there attention for defining clear roles and responsibilities for all? 2. Is there attention for defining clear decision-making structures for all? 3. Is there attention for including specific tools (e.g. roles and responsibility charts, procedural guidance documents, protocols) and mechanisms (e.g. meetings, workshops, training sessions) to ensure that all participants understand their own and others’ roles and responsibilities? 4. Is there attention for clear documentation and explanation of available funding resources prior to involvement? 5. Is there attention for communicating any changes that could occur during the PE initiative up-front? 6. Is there attention for sharing the outcomes of the PE initiative with all the stakeholders using appropriate channels and formats suited to patient perspectives’ circumstances and needs? | 8a Yes, there is attention for clearly defining roles and responsibilities for all.  8b No, there is no attention for defining roles and responsibilities for all.  8c It is not possible to assess based on the available information.  8d This question is not relevant to this initiative.  9a Yes, there is attention for clearly decision-making structures for all.  9b No, there is no attention for defining decision-making structures for all.  9c It is not possible to assess based on the available information.  9d This question is not relevant to this initiative.  10a Yes, there is attention for including specific tools and mechanisms.  10b. Yes, there is attention for including specific tools or mechanisms, but not for both.  10c No, there is no attention for including tools and mechanisms.  10d It is not possible to assess based on the available information.  10e This question is not relevant to this initiative.  11a Yes, there is attention for documentation and explanation of funding resources.  11b No, there is no attention for documentation and explanation of funding resources.  11c It is not possible to assess based on the available information.  11d This question is not relevant to this initiative.  12a Yes, there is attention for communicating changes that could occur during the PE initiative up-front  12b No, there is no attention for communicating changes that could occur during the PE initiative.  12c It is not possible to assess based on the available information.  12d This question is not relevant to this initiative.  13a Yes, there is attention for sharing outcomes with all the stakeholders using appropriate channels and formats.  13b Yes, there is attention for sharing outcomes with all the stakeholders, but there is no attention for using appropriate channels and formats.  13c No, there is no attention for sharing outcomes with all the stakeholders  13d It is not possible to assess based on the available information.  13e This question is not relevant to this initiative. | 1. Were roles and responsibilities clearly defined for all? 2. Were decision-making structures known to all stakeholders involved? 3. Did the initiative include specific tools (e.g. roles and responsibility charts, procedural guidance documents, protocols) and mechanisms (e.g. meetings, workshops, training sessions) to ensure that all participants understand their own and others’ roles and responsibilities? 4. Did the initiative clearly document and explain available funding resources prior to involvement? 5. Did the initiative communicate any changes that occurred during the PE initiative up-front? 6. Were the outcomes of the PE initiative shared with all the stakeholders using appropriate channels and formats suited to patient perspectives’ circumstances and needs? | 8a Yes, roles and responsibilities were clearly defined for all.  8b No roles and responsibilities were defined for all.  8c It is not possible to assess based on the available information.  8d This question is not relevant to this initiative.  9a Yes, decision-making structures were clearly defined for all.  8b No decision-making structures were defined for all.  9c It is not possible to assess based on the available information.  9d This question is not relevant to this initiative.  10a Yes, specific tools and mechanisms were included.  10b. Yes specific tools or mechanisms were included but not both.  10c No tools and mechanisms are included.  10d It is not possible to assess based on the available information.  10e This question is not relevant to this initiative.  11a Yes, funding resources were clearly documented and explained.  11b No, funding resources were not documented and/or explained.  11c It is not possible to assess based on the available information.  12d This question is not relevant to this initiative.  12a Yes, changes that occurred during the PE initiative were communicated up-front  12b No, changes that occurred during the PE initiative were not communicated.  12c It is not possible to assess based on the available information.  12d This question is not relevant to this initiative.  13a Yes, outcomes were shared with all the stakeholders using appropriate channels and formats.  13b Yes, outcomes were shared with all the stakeholders, but appropriate channels and formats were not used.  13c No, outcomes were not shared with all the stakeholders  13d It is not possible to assess based on the available information.  13e This question is not relevant to this initiative. |
|  |  |  |  | **FREE TEXT OPTION (MAX 250 WORDS)**  If the answer was “NO” or “Not possible to assess” or if the question is “Not considered relevant to that specific initiative” to the previous questions please provide additional specific information. Examples include: limited detail in the report, lack of clarity, or ambiguity of the ‘who’, ’what’, ’when’ and ‘how’.  Please also describe what other “gaps” or aspects of the gap need to be considered.  -Why information on criteria ‘X’ was not available?  -Why criteria ‘X’ or ‘Y’ was not undertaken, considered, or followed? | | **FREE TEXT OPTION (MAX 250 WORDS)**  If the answer was “NO” or “Not possible to assess” or if the question is “Not considered relevant to that specific initiative” to the previous questions please provide additional specific information. Examples include: limited detail in the report, lack of clarity, or ambiguity of the ‘who’, ’what’, ’when’ and ‘how’.  Please also describe what other “gaps” or aspects of the gap need to be considered.  -Why information on criteria ‘X’ was not available?  -Why criteria ‘X’ or ‘Y’ was not undertaken, considered, or followed? | |
|  |  | Communication & feedback | - **Regular communication** occurred throughout - **Feedback and outcomes were communicated in a clear and adapted way**, using plain language, accessible formats, and taking into account potential disabilities and impairments, as appropriate. - A **named key contact** (up-to-date and single point of contact) that patients could reach out throughout the process for information and/or support was made available - All participants were given the opportunity **to give** **regular feedback.** - **All legal agreements** (including the **terms and condition) were written and communicated** in a clear and accessible way and adapted to the target population involved **A co created dissemination and communication plan** for sharing the process and outcomes was in place | 1. Is there attention for regular communication whenever appropriate about the initiative? 2. Is there attention for communicating feedback and outcomes in a clear and adapted way (e.g. using plain language, accessible formats), and taking into account potential disabilities and impairments, as appropriate? 3. Is there attention for providing a named a key contact (up-to-date and single point of contact) to patients to reach out to throughout the process for information and/or support? 4. Is there attention to giving participants the opportunity to give regular feedback? 5. Is there attention for providing all legal agreements (including terms and conditions) written in a clear and accessible way and adapted to the target population involved? 6. Is there attention for including a dissemination and communication plan sharing the process and outcomes? | 14a Yes, there is attention for regular communication whenever appropriate about the initiative.  14b No, there is no attention for communication about the initiative.  14c It is not possible to assess based on the available information.  14d This question is not relevant to this initiative.  15a Yes, there is attention for communicating feedback and outcomes in a clear and adapted way.  15b No, no there is no attention for communicating feedback and outcomes in a clear and adapted way.  15c It is not possible to assess based on the available information.  15d This question is not relevant to this initiative.  16a Yes, there is attention for providing a named key contact to patients.  16b No, there is no attention for providing a named key contact to patients.  16c It is not possible to assess based on the available information.  16d This question is not relevant to this initiative.  17a Yes, there is attention to giving all participants the opportunity to give regular feedback.  17b No, there is no attention for giving participants the opportunity to give regular feedback.  17c It is not possible to assess based on the available information.  17d This question is not relevant to this initiative.  18a Yes, there is attention for providing all legal agreements written in a clear and accessible way and adapted to the target population involved.  18b Yes, there is attention for providing legal agreements, but there is no attention for writing it in a clear and accessible way or adapt it to the target population involved.  18c No, there is no attention for providing legal agreements.  18d It is not possible to assess based on the available information.  18e This question is not relevant to this initiative.  19a Yes, there is attention for including a dissemination and communication plan sharing the process and outcomes.  19b No, there is no attention for including a dissemination and communication plan for sharing the process and outcomes.  19c It is not possible to assess based on the available information.  19d This question is not relevant to this initiative. | 1. Did regular communication occur whenever appropriate about the initiative? 2. Did the initiative communicate feedback and outcomes in a clear and adapted way (e.g. using plain language, accessible formats), and taking into account potential disabilities and impairments, as appropriate? 3. Was a named key contact (up-to-date and single point of contact) provided to patients to reach out to throughout the process for information and/or support? 4. Were all participants given the opportunity to give regular feedback? 5. Were all legal agreements (including terms and conditions) written in a clear and accessible way and adapted to the target population involved? 6. Did the initiative include a dissemination and communication plan sharing the process and outcomes? | 14a Yes, regular communication occurred whenever appropriate about the initiative.  14b No communication occurred about the initiative.  14c It is not possible to assess based on the available information.  14d This question is not relevant to this initiative.  15a Yes, feedback and outcomes were communicated in a clear and adapted way.  15b No, feedback and outcomes were not communicated in a clear and adapted way.  15c It is not possible to assess based on the available information.  15d This question is not relevant to this initiative.  16a Yes, a named key contact was provided to patients.  16b No, a named key contact was not provided to patients.  16c It is not possible to assess based on the available information.  16d This question is not relevant to this initiative.  17a Yes, all participants were given the opportunity to give regular feedback.  17b No, participants were not given the opportunity to give regular feedback.  17c It is not possible to assess based on the available information.  17d This question is not relevant to this initiative.  18a Yes, all legal agreements were written in a clear and accessible way and adapted to the target population involved.  18b Yes, legal agreements were written but not in a clear and accessible way or adapted to the target population involved.  18c No, legal agreements were not written and included.  18d It is not possible to assess based on the available information.  18e This question is not relevant to this initiative.  19a Yes, a dissemination and communication plan sharing the process and outcomes was included.  19b No, a dissemination and communication plan for sharing the process and outcomes was not included.  19c It is not possible to assess based on the available information.  19d This question is not relevant to this initiative. |
|  |  |  |  | **FREE TEXT OPTION (MAX 250 WORDS)**  If the answer was “NO” or “Not possible to assess” or if the question is “Not considered relevant to that specific initiative” to the previous questions please provide additional specific information. Examples include: limited detail in the report, lack of clarity, or ambiguity of the ‘who’, ’what’, ’when’ and ‘how’.  Please also describe what other “gaps” or aspects of the gap need to be considered.  -Why information on criteria ‘X’ was not available?  -Why criteria ‘X’ or ‘Y’ was not undertaken, considered, or followed? | | **FREE TEXT OPTION (MAX 250 WORDS)**  If the answer was “NO” or “Not possible to assess” or if the question is “Not considered relevant to that specific initiative” to the previous questions please provide additional specific information. Examples include: limited detail in the report, lack of clarity, or ambiguity of the ‘who’, ’what’, ’when’ and ‘how’.  Please also describe what other “gaps” or aspects of the gap need to be considered.  -Why information on criteria ‘X’ was not available?  -Why criteria ‘X’ or ‘Y’ was not undertaken, considered, or followed? | |
|  |  | Feasibility of collaboration and timing of involvement | **Mechanisms are in place** (e.g. language, format of meetings, the venue, time of the day etc.) **to ensure participation of patient representatives**, taking into consideration participant’s circumstances, linked to but not limited to possible physical or mental impairments, cultural background, age and other relevant features (e.g. recordings, virtual communication, use of language, format of meetings, the venue, and information provided).   - The schedule and timelines involved respect the need for **planning and preparation time**, thereby allowing patient representatives to effectively engage from the beginning and throughout the process. - There is **involvement from start until completion**. | 1. Is there attention for including mechanisms (e.g. language, format of meetings, the venue, time of the day etc.) to ensure participation of patient representatives, taking into consideration participant’s circumstances, linked to but not limited to possible physical or mental impairments, cultural background, age and other relevant features (e.g. recordings, virtual communication, use of language, format of meetings, the venue, and information provided)? 2. Is there attention for the need for planning and preparation time in the schedule and timeline, thereby allowing patient representatives to effectively engage from the beginning and throughout the process? 3. Is there attention for engaging patients from start until completion? | 20a Yes, there is attention for including mechanisms to ensure participation taking into account participants’ personal circumstances.  20b No, there is no attention for including mechanisms to ensure participation taking into account participants’ personal circumstances.  20c It is not possible to assess based on the available information.  20d This question is not relevant to this initiative.  21a Yes, there is attention for the need for planning and preparation time in the schedule and timeline.  21b No, there is no attention for the need for planning and preparation time in the schedule and timeline.  21c It is not possible to assess based on the available information.  21d This question is not relevant to this initiative.  22a Yes, there is attention for engaging patients from start until completion.  22b No, there is no attention for engaging patients from start until completion.  22c It is not possible to assess based on the available information.  22d This question is not relevant to this initiative. | 1. Were there mechanisms (e.g. language, format of meetings, the venue, time of the day etc.) in place to ensure participation of patient representatives, taking into consideration participant’s circumstances, linked to but not limited to possible physical or mental impairments, cultural background, age and other relevant features (e.g. recordings, virtual communication, use of language, format of meetings, the venue, and information provided)? 2. Did the schedule and timeline respect the need for planning and preparation time, thereby allowing patient representatives to effectively engage from the beginning and throughout the process? 3. Were patients engaged from start until completion? | 209a Yes, mechanisms were in place to ensure participation taking into account participants’ personal circumstances.  20b No, mechanisms were not in place to ensure participation taking into account participants’ personal circumstances.  1920c It is not possible to assess based on the available information.  20d This question is not relevant to this initiative.  21a Yes, the schedule and timeline respected the need for planning and preparation time.  21b No, the schedule and timeline did not respected the need for planning and preparation time.  21c It is not possible to assess based on the available information.  21d This question is not relevant to this initiative.  22a Yes, patients were engaged from start until completion.  22b No, patients were not engaged from start until completion.  22c It is not possible to assess based on the available information.  22d This question is not relevant to this initiative. |
|  |  |  |  | **FREE TEXT OPTION (MAX 250 WORDS)**  If the answer was “NO” or “Not possible to assess” or if the question is “Not considered relevant to that specific initiative” to the previous questions please provide additional specific information. Examples include: limited detail in the report, lack of clarity, or ambiguity of the ‘who’, ’what’, ’when’ and ‘how’.  Please also describe what other “gaps” or aspects of the gap need to be considered.  -Why information on criteria ‘X’ was not available?  -Why criteria ‘X’ or ‘Y’ was not undertaken, considered, or followed? | | **FREE TEXT OPTION (MAX 250 WORDS)**  If the answer was “NO” or “Not possible to assess” or if the question is “Not considered relevant to that specific initiative” to the previous questions please provide additional specific information. Examples include: limited detail in the report, lack of clarity, or ambiguity of the ‘who’, ’what’, ’when’ and ‘how’.  Please also describe what other “gaps” or aspects of the gap need to be considered.  -Why information on criteria ‘X’ was not available?  -Why criteria ‘X’ or ‘Y’ was not undertaken, considered, or followed? | |
|  |  | Sustainability | - The PE initiative is **embedded in the institution or organization** and when relevant, the alliances with private and/or other public institution(s) to ensure continuity. - **Human and financial resources** are allocated for the long-term continuity and implementation of outcomes - **Formation and maintenance of a partnership between all stakeholders** to ensure continuity | 1. Is there attention for embedding the initiative in the institution or organization and, when relevant, is there attention for alliances with private and/or other public institution(s) to ensure continuity? 2. Is there attention for allocating human and financial resources for the long-term continuity of the PE initiative? 3. Is the attention for ensuring the formation and maintenance of a partnership between all stakeholders to ensure continuity? | 23a Yes, there is attention for embedding the initiative in the institution or organization.  23b No, there is no attention for embedding the initiative in the institution or organization  23c It is not possible to assess based on the available information.  23d This question is not relevant to this initiative.  24a Yes, there is attention for human and financial resources for the long-term continuity of the practice on PE  24b No, there is no attention for allocating human and financial resources for the long-term continuity of the PE initiative.  24c It is not possible to assess based on the available information.  24d This question is not relevant to this initiative.  25a Yes, there is attention for ensuring the formation and maintenance of partnerships.  25b No, there is no attention for ensuring the formation and maintenance of partnerships.  25c It is not possible to assess based on the available information.  25d This question is not relevant to this initiative. | 1. Was the initiative embedded in the institution or organization and, when relevant, were there alliances with private and/or other public institution(s) to ensure continuity? 2. Did the organization responsible for the initiative allocate human and financial resources for the long-term continuity of the PE initiative? 3. Did the initiative ensure the formation and maintenance of a partnership between all stakeholders to ensure continuity? | 23a Yes, the initiative was embedded in the institution or organization.  23b No, the initiative was not embedded in the institution or organization  23c It is not possible to assess based on the available information.  23d This question is not relevant to this initiative.  24a Yes, the organization responsible for the initiative allocated human and financial resources for the long-term continuity of the practice on PE  24b No, the organization responsible for the initiative did not allocate human and financial resources for the long-term continuity of the PE initiative.  24c It is not possible to assess based on the available information.  24d This question is not relevant to this initiative.  25a Yes, the initiative ensured the formation and maintenance of partnerships  25b No, the initiative did not ensure the formation and maintenance of a partnership  25c It is not possible to assess based on the available information.  25d This question is not relevant to this initiative. |
|  |  |  |  | **FREE TEXT OPTION (MAX 250 WORDS)**  If the answer was “NO” or “Not possible to assess” or if the question is “Not considered relevant to that specific initiative” to the previous questions please provide additional specific information. Examples include: limited detail in the report, lack of clarity, or ambiguity of the ‘who’, ’what’, ’when’ and ‘how’.  Please also describe what other “gaps” or aspects of the gap need to be considered.  -Why information on criteria ‘X’ was not available?  -Why criteria ‘X’ or ‘Y’ was not undertaken, considered, or followed? | | **FREE TEXT OPTION (MAX 250 WORDS)**  If the answer was “NO” or “Not possible to assess” or if the question is “Not considered relevant to that specific initiative” to the previous questions please provide additional specific information. Examples include: limited detail in the report, lack of clarity, or ambiguity of the ‘who’, ’what’, ’when’ and ‘how’.  Please also describe what other “gaps” or aspects of the gap need to be considered.  -Why information on criteria ‘X’ was not available?  -Why criteria ‘X’ or ‘Y’ was not undertaken, considered, or followed? | |
|  | **Process management** | Equal treatment of participants | - **Mechanisms are in place** (e.g. neutral facilitation, open and respectful atmosphere, mutual respect) **to ensure a fair deliberative process** that allows equal opportunity for all participants’ contribution. | 1. Is there attention for mechanisms (e.g. neutral facilitation, open and respectful atmosphere, mutual respect) to ensure a fair deliberative process that allows equal opportunity for all participants’ contribution? | 26a Yes, there is attention for mechanisms to ensure a fair deliberative process  26b No, there is no attention for mechanisms to ensure a fair deliberative process.  26c It is not possible to assess based on the available information.  26d This question is not relevant to this initiative. | 1. Were there mechanisms (e.g. neutral facilitation, open and respectful atmosphere, mutual respect) to ensure a fair deliberative process that allows equal opportunity for all participants’ contribution? | 26a Yes, mechanisms were in place to ensure a fair deliberative process  26b No, mechanisms were not in place to ensure a fair deliberative process.  26c It is not possible to assess based on the available information.  26d This question is not relevant to this initiative. |
|  |  |  |  | **FREE TEXT OPTION (MAX 250 WORDS)**  If the answer was “NO” or “Not possible to assess” or if the question is “Not considered relevant to that specific initiative” to the previous questions please provide additional specific information. Examples include: limited detail in the report, lack of clarity, or ambiguity of the ‘who’, ’what’, ’when’ and ‘how’.  Please also describe what other “gaps” or aspects of the gap need to be considered.  -Why information on criteria ‘X’ was not available?  -Why criteria ‘X’ or ‘Y’ was not undertaken, considered, or followed? | | **FREE TEXT OPTION (MAX 250 WORDS)**  If the answer was “NO” or “Not possible to assess” or if the question is “Not considered relevant to that specific initiative” to the previous questions please provide additional specific information. Examples include: limited detail in the report, lack of clarity, or ambiguity of the ‘who’, ’what’, ’when’ and ‘how’.  Please also describe what other “gaps” or aspects of the gap need to be considered.  -Why information on criteria ‘X’ was not available?  -Why criteria ‘X’ or ‘Y’ was not undertaken, considered, or followed? | |
|  |  | Legal & ethical considerations | - A **code of conduct**, which clearly states the (ethical) principles, governance requirement, rules and procedure of participation for all stakeholders involved is in place - A privacy policy is in place which describes policy to maintain data privacy of engaged patient in the engagement. - Procedures to **identify and address potential discriminatory, coercive, intimidating, and unethical behaviours**, towards all stakeholders are in place for, before, during and after their participation*.* - **Potential conflicts of interest are addressed and managed** (up to avoidance). For this, policies that require full disclosure, transparency and accountability are developed - The **terms and conditions of all policies and confidentiality agreements are in place**, presented in a clear and accessible way to the target stakeholders involved. | 1. Is there attention for a code of conduct, which clearly states the (ethical) principles, governance requirement, rules and procedure of participation for all stakeholders involved? 2. Is there attention for a privacy policy that describes policy to maintain data privacy of engaged patients in the engagement? 3. Is there attention for the identification and addressment of potential discriminatory, coercive, intimidating, and unethical behaviours, towards all stakeholders, before, during and after their participation*?* 4. Is there attention for the management of potential conflicts of interest (up to avoidance)? For this, policies that require full disclosure, transparency and accountability should be developed. 5. Is there attention for the presentation of the terms and conditions of all policies and confidentiality agreements, in a clear and accessible way to the stakeholders involved? | 27a Yes, there is attention for a code of conduct  27b No, there is no attention for a code of conduct  27c It is not possible to assess based on the available information.  27d This question is not relevant to this initiative.  28a Yes, there is attention for a privacy policy  28b No, there is no attention for a privacy policy  28c It is not possible to assess based on the available information.  28d This question is not relevant to this initiative.  29a Yes, there is attention for procedures to identify and address potential discriminatory, coercive, intimidating, and unethical behaviours  29b No, there is no attention for procedures to identify and address potential discriminatory, coercive, intimidating, and unethical behaviors  29c It is not possible to assess based on the available information.  29d This question is not relevant to this initiative.  30a Yes, there is attention for potential conflicts of interest  30b No, the is no attention for potential conflicts of interest  30c It is not possible to assess based on the available information.  30d This question is not relevant to this initiative.  31a Yes, there is attention for the presentation of all terms and conditions of policies and confidentiality agreements, in a clear and accessible way  31b No, there is no attention for the presentation of the terms and conditions of policies and confidentiality agreements in a clear and accessible way  31c It is not possible to assess based on the available information.  31d This question is not relevant to this initiative. | 1. Was there a code of conduct, which clearly states the (ethical) principles, governance requirement, rules and procedure of participation for all stakeholders involved in place? 2. Was there a privacy policy, which clearly describes a policy to maintain privacy of engaged patients? 3. Were there procedures in place to identify and address potential discriminatory, coercive, intimidating, and unethical behaviours, towards all stakeholders, before, during and after their participation*?* 4. Were potential conflicts of interest (up to avoidance) addressed and managed? For this, policies that require full disclosure, transparency and accountability developed. 5. Were the terms and conditions of all policies and confidentiality agreements, presented in a clear and accessible way to the target population involved? | 27a Yes, a code of conduct was in place  27b No, a code of conduct was not in place  27c It is not possible to assess based on the available information.  27d This question is not relevant to this initiative.  28a Yes, a privacy policy was in place  28b No, a privacy policy was not in place  28c It is not possible to assess based on the available information.  28d This question is not relevant to this initiative.  29a Yes, procedures were in place to identify and address potential discriminatory, coercive, intimidating, and unethical behaviors  29b No, procedures were not in place to identify and address potential discriminatory, coercive, intimidating, and unethical behaviours  29c It is not possible to assess based on the available information.  29d This question is not relevant to this initiative.  30a Yes, potential conflicts of interest were addressed and managed  30b No, potential conflicts of interest were not addressed and managed  30c It is not possible to assess based on the available information.  30d This question is not relevant to this initiative.  31a Yes, all terms and conditions of policies and confidentiality agreements were presented in a clear and accessible way  31b No, terms and conditions of policies and confidentiality agreements were not presented in a clear and accessible way  31c It is not possible to assess based on the available information.  31d This question is not relevant to this initiative. |
|  |  |  |  | **FREE TEXT OPTION (MAX 250 WORDS)**  If the answer was “NO” or “Not possible to assess” or if the question is “Not considered relevant to that specific initiative” to the previous questions please provide additional specific information. Examples include: limited detail in the report, lack of clarity, or ambiguity of the ‘who’, ’what’, ’when’ and ‘how’.  Please also describe what other “gaps” or aspects of the gap need to be considered.  -Why information on criteria ‘X’ was not available?  -Why criteria ‘X’ or ‘Y’ was not undertaken, considered, or followed? | | **FREE TEXT OPTION (MAX 250 WORDS)**  If the answer was “NO” or “Not possible to assess” or if the question is “Not considered relevant to that specific initiative” to the previous questions please provide additional specific information. Examples include: limited detail in the report, lack of clarity, or ambiguity of the ‘who’, ’what’, ’when’ and ‘how’.  Please also describe what other “gaps” or aspects of the gap need to be considered.  -Why information on criteria ‘X’ was not available?  -Why criteria ‘X’ or ‘Y’ was not undertaken, considered, or followed? | |
|  |  | Supportive Resources | - A clear, transparent and equitable (fair) **financial compensation framework** is in place and made available for patient representatives* (e.g. reimbursement of expenses for travel, time missed from work, subsistence, child/elderly care, stipends) - **Sufficient funding is allocated** to cover governance, administration and relevant operations | 32. Is there attention for a clear, transparent and equitable (fair) financial compensation framework to be in place and made available for patient representatives* who participate (e.g. reimbursement of expenses for travel, time missed from work, subsistence, child/elderly care, stipends)?  33. Is there attention for the allocation of sufficient funding to cover governance, administration and relevant operations? | 32a Yes, there is attention for a clear, transparent and equitable (fair) financial compensation framework.  32b Yes, there is attention for a financial compensation framework, but no special attention for transparency and equitability.  32c No, there is no attention for a clear, transparent and equitable (fair) financial compensation framework  32d It is not possible to assess based on the available information.  32e This question is not relevant to this initiative.  33a Yes, there is attention for the allocation of sufficient funding to cover governance, administration and relevant operations  33b No, there is no attention for the allocation of sufficient funding to cover governance, administration and relevant operations  33c It is not possible to assess based on the available information.  33d This question is not relevant to this initiative. | 1. Was there a clear, transparent and equitable (fair) financial compensation framework in place and made available for patient representatives* who participate (e.g. reimbursement of expenses for travel, time missed from work, subsistence, child/elderly care, stipends)? 2. Did the initiative allocate sufficient funding to cover governance, administration and relevant operations? | 32a Yes, a clear, transparent and equitable (fair) financial compensation framework was in place.  32b Yes, a financial compensation framework was in place, but it was not transparent and equitable.  32c No, a clear, transparent and equitable (fair) financial compensation framework was not in place  32d It is not possible to assess based on the available information.  32e This question is not relevant to this initiative.  33a Yes, sufficient funding to cover governance, administration and relevant operations was in place  33b No, sufficient funding to cover governance, administration and relevant operations was not in place  33c It is not possible to assess based on the available information.  33d This question is not relevant to this initiative. |
|  |  |  |  | **FREE TEXT OPTION (MAX 250 WORDS)**  If the answer was “NO” or “Not possible to assess” or if the question is “Not considered relevant to that specific initiative” to the previous questions please provide additional specific information. Examples include: limited detail in the report, lack of clarity, or ambiguity of the ‘who’, ’what’, ’when’ and ‘how’.  Please also describe what other “gaps” or aspects of the gap need to be considered.  -Why information on criteria ‘X’ was not available?  -Why criteria ‘X’ or ‘Y’ was not undertaken, considered, or followed? | | **FREE TEXT OPTION (MAX 250 WORDS)**  If the answer was “NO” or “Not possible to assess” or if the question is “Not considered relevant to that specific initiative” to the previous questions please provide additional specific information. Examples include: limited detail in the report, lack of clarity, or ambiguity of the ‘who’, ’what’, ’when’ and ‘how’.  Please also describe what other “gaps” or aspects of the gap need to be considered.  -Why information on criteria ‘X’ was not available?  -Why criteria ‘X’ or ‘Y’ was not undertaken, considered, or followed? | |
|  | **Learning & changes**  **Outcome criteria** | Direct outcomes | - **Outcomes measures were related to the aims and objectives** of the initiative. - **Reflection of patients’ perspective are clearly defined in the outcomes / result**. - **Outcomes / results demonstrate a consensus by all participants** - Achieved **(mutual) learning on substantive matters** | 1. Is there attention for the documentation of the outcomes of the initiative? 2. Is there attention for consensus among participants on the outcomes? 3. Is there attention for mutual learning among stakeholders? | 34a No attention for the documentation of the outcomes of the initiative. The questionnaire finishes here.  34b Yes, there is attention for the documentation of the outcomes of the initiative.  34c It is not possible to assess based on the available information.  34d This question is not relevant to this initiative.  [If YES please answer in the following questions (Q33-35), otherwise skip to Q36].  35.a Yes, there is attention for consensus among participants on the outcomes  35.b No, there is no attention for consensus among participants on the outcomes  35c It is not possible to assess based on the available information.  35d This question is not relevant to this initiative.  36a Yes, there is attention for mutual learning among stakeholders.  26b No, there is no attention for mutual learning among stakeholders.  36c It is not possible to assess based on the available information.  36d This question is not relevant to this initiative. | 34. Were outcomes of the initiative documented?  35. Was there consensus among participants on the outcomes?  36. Did the initiative result in mutual learning among stakeholders? | 32a No outcomes were documented. The questionnaire finishes here.  32b Yes, outcomes were documented.  32c It is not possible to assess based on the available information.  32d This question is not relevant to this initiative.  [If YES please answer in the following questions (Q33-35), otherwise skip to Q36].  35.a Yes, there was consensus among participants on the outcomes  35.b No, there was no consensus among participants on the outcomes  35c It is not possible to assess based on the available information.  35d This question is not relevant to this initiative.  36a Yes, the initiative resulted in mutual learning among stakeholders.  36b No, the initiative did not result in mutual learning among stakeholders.  36c It is not possible to assess based on the available information.  36d This question is not relevant to this initiative. |
|  |  |  |  | **FREE TEXT OPTION (MAX 250 WORDS)**  If the answer was “NO” or “Not possible to assess” or if the question is “Not considered relevant to that specific initiative” to the previous questions please provide additional specific information. Examples include: limited detail in the report, lack of clarity, or ambiguity of the ‘who’, ’what’, ’when’ and ‘how’.  Please also describe what other “gaps” or aspects of the gap need to be considered.  -Why information on criteria ‘X’ was not available?  -Why criteria ‘X’ or ‘Y’ was not undertaken, considered, or followed? | | **FREE TEXT OPTION (MAX 250 WORDS)**  If the answer was “NO” or “Not possible to assess” or if the question is “Not considered relevant to that specific initiative” to the previous questions please provide additional specific information. Examples include: limited detail in the report, lack of clarity, or ambiguity of the ‘who’, ’what’, ’when’ and ‘how’.  Please also describe what other “gaps” or aspects of the gap need to be considered.  -Why information on criteria ‘X’ was not available?  -Why criteria ‘X’ or ‘Y’ was not undertaken, considered, or followed? | |
|  |  | Impact for R&D | - **Feedback on the implementation of outcomes** in practice - **Use of metrics to measure impact** of patient engagement. | 37. Is there attention for the implementation of the outcomes in practice?  38. Does the initiative propose metrics to measure impact of PE? If yes, which metrics are proposed? …. | 37a Yes, there is attention for the implementation of the outcomes in practice  37b No, there is no attention for the implementation of the outcomes in practice  37c It is not possible to assess based on the available information.  37d This question is not relevant to this initiative.  38a Yes, metrics are proposed to measure the impact.  38b No, metrics are not proposed to measure the impact.  38c It is not possible to assess based on the available information.  38d This question is not relevant to this initiative. | 1. Were the outcomes implemented in practice? 2. Did the initiative use metrics to measure impact of PE? th | 37.a Yes, outcomes were implemented in practice  37.b No, outcomes were not implemented in practice  37c It is not possible to assess based on the available information.  37d This question is not relevant to this initiative.  38a Yes, metrics were used to measure the impact. Please specify the metrics…  38b No, metrics were not used to measure the impact  38c It is not possible to assess based on the available information.  38d This question is not relevant to this initiative. |
|  |  |  |  | **FREE TEXT OPTION (MAX 250 WORDS)**  If the answer was “NO” or “Not possible to assess” or if the question is “Not considered relevant to that specific initiative” to the previous questions please provide additional specific information. Examples include: limited detail in the report, lack of clarity, or ambiguity of the ‘who’, ’what’, ’when’ and ‘how’.  Please also describe what other “gaps” or aspects of the gap need to be considered.  -Why information on criteria ‘X’ was not available?  -Why criteria ‘X’ or ‘Y’ was not undertaken, considered, or followed? | | **FREE TEXT OPTION (MAX 250 WORDS)**  If the answer was “NO” or “Not possible to assess” or if the question is “Not considered relevant to that specific initiative” to the previous questions please provide additional specific information. Examples include: limited detail in the report, lack of clarity, or ambiguity of the ‘who’, ’what’, ’when’ and ‘how’.  Please also describe what other “gaps” or aspects of the gap need to be considered.  -Why information on criteria ‘X’ was not available?  -Why criteria ‘X’ or ‘Y’ was not undertaken, considered, or followed? | |
|  |  | Value of PE for stakeholders | - Evidence of value is captured and reported | 1. Is there attention for the documentation of the value of the initiative for different stakeholder groups? | 39a. Yes, there is attention for the documentation of the value of the initiative for different stakeholder groups.  39b. No, there is no attention for the documentation of the value of the initiative for different stakeholder groups.  39c It is not possible to assess based on the available information.  39d This question is not relevant to this initiative.  If yes, tick all stakeholders group to which there is attention:   - 1. Patient representatives   2. Regulators   3. HTA   4. Industry   5. Other, please specify:   6. It is not possible to assess based on the available information   **FREE TEXT OPTION (MAX 250 WORDS)**  Please provide detail of available evidence | 1. Was there any evidence of value provided and if so for which stakeholder group | 39a. Yes, there is evidence of value provided.  39b. No, there is no evidence of value provided.  39c It is not possible to assess based on the available information.  39d This question is not relevant to this initiative.  If yes, tick all stakeholders group to which evidence of value is provided.   - 1. Patient representatives   2. Regulators   3. HTA   4. Industry   5. Other, please specify:   6. It is not possible to assess based on the available information   **FREE TEXT OPTION (MAX 250 WORDS)**  Please provide detail of available evidence. |
|  |  |  |  | **FREE TEXT OPTION (MAX 250 WORDS)**  If the answer was “NO” or “Not possible to assess” or if the question is “Not considered relevant to that specific initiative” to the previous questions please provide additional specific information. Examples include: limited detail in the report, lack of clarity, or ambiguity of the ‘who’, ’what’, ’when’ and ‘how’.  Please also describe what other “gaps” or aspects of the gap need to be considered.  -Why information on criteria ‘X’ was not available?  -Why criteria ‘X’ or ‘Y’ was not undertaken, considered, or followed? | | **FREE TEXT OPTION (MAX 250 WORDS)**  If the answer was “NO” or “Not possible to assess” or if the question is “Not considered relevant to that specific initiative” to the previous questions please provide additional specific information. Examples include: limited detail in the report, lack of clarity, or ambiguity of the ‘who’, ’what’, ’when’ and ‘how’.  Please also describe what other “gaps” or aspects of the gap need to be considered.  -Why information on criteria ‘X’ was not available?  -Why criteria ‘X’ or ‘Y’ was not undertaken, considered, or followed? | |
|  |  | Learning & reflection on the PE practice | - There are **methods, tools and monitoring systems in place to evaluate the PE practice** systematically and at appropriate phases of the process. - The **evaluation outcomes are used to improve future PE practices***.* - The **evaluation framework is included and shared** among the participants - **The evaluation criteria are linked to the aims and objectives** of the PE practice. | 1. Is there attention for learning and reflection on the PE practice? 2. Does the initiative propose methods, tools and monitoring systems to evaluate the PE practice systematically and at appropriate phases of the process? 3. Does the initiative propose an evaluation framework for evaluation of the PE initiative? 4. Is there attention for the link between the evaluation criteria and the aims and practices of the PE practice?   Is there attention for the use of the evaluation outcomes to improve future PE initiatives? | 40a Yes there is attention for learning and reflection on the PE practice.  40b. No, there is no attention for learning and reflection on the PE practice. The questionnaire finishes here.  40c It is not possible to assess based on the available information.  40d This question is not relevant to this initiative.  41a Yes, there are methods, tools and monitoring systems proposed to evaluate the PE practice systematically and at appropriate phases of the process.  41b No, there are no methods, tools and monitoring systems proposed to evaluate the PE practice systematically and at appropriate phases of the process.  41c It is not possible to assess based on the available information.  41d This question is not relevant to this initiative.  [If YES please answer the following questions (Q40-43), otherwise skip to END].  42a Yes, an evaluation framework for evaluation of the PE initiative is proposed.  42b No an evaluation framework for evaluation of the PE initiative is not proposed.  42c It is not possible to assess based on the available information.  42d This question is not relevant to this initiative.  43a Yes, there is attention for the link between the evaluation criteria and the aims and practices of this PE initiative  43b No, there is no attention for the link between the evaluation criteria and the aims and practices of this PE initiative  43c It is not possible to assess based on the available information.  43d This question is not relevant to this initiative.  44a Yes, there is attention for the use of the evaluation outcomes to improve future PE initiatives.  44b No, there is no attention for the use of the evaluation outcomes to improve future PE initiatives.  44c It is not possible to assess based on the available information.  44d This question is not relevant to this initiative. | 1. Was there attention for learning & reflection on the PE practice 2. Did the initiative have methods, tools and monitoring systems in place to evaluate the PE practice systematically and at appropriate phases of the process? 3. Did the initiative include and share an evaluation framework for evaluation of the PE initiative? 4. Were the evaluation criteria linked to the aims and practices of this PE initiative? 5. Were the evaluation outcomes used to improve future PE initiatives? | 40a Yes there was attention for learning and reflection on the PE practice.  40b. No, there was no attention for learning and reflection on the PE practice. The questionnaire finishes here.  40c It is not possible to assess based on the available information.  40d This question is not relevant to this initiative.  41a Yes, there are methods, tools and monitoring systems in place to evaluate the PE practice systematically and at appropriate phases of the process.  41b No, there are no methods, tools and monitoring systems in place to evaluate the PE practice systematically and at appropriate phases of the process.  41c It is not possible to assess based on the available information.  41d This question is not relevant to this initiative.  [If YES please answer the following questions (Q40-43), otherwise skip to END].  42a Yes, the initiative includes and shares and evaluation framework for evaluation of the PE initiative.  42b No, the initiative does not include and share and evaluation framework for evaluation of the PE initiative.  42c It is not possible to assess based on the available information.  42d This question is not relevant to this initiative.  43a Yes, evaluation criteria are linked to the aims and practices of PE.  43b No, evaluation criteria are not linked to the aims and practices of this PE initiative.  43c It is not possible to assess based on the available information.  43d This question is not relevant to this initiative.  44a Yes, evaluation outcomes are used to improve future PE initiatives.  44b No, evaluation outcomes are not used to improve future PE initiatives.  44c It is not possible to assess based on the available information.  44d This question is not relevant to this initiative. |
|  |  |  |  | **FREE TEXT OPTION (MAX 250 WORDS)**  If the answer was “NO” or “Not possible to assess” or if the question is “Not considered relevant to that specific initiative” to the previous questions please provide additional specific information. Examples include: limited detail in the report, lack of clarity, or ambiguity of the ‘who’, ’what’, ’when’ and ‘how’.  Please also describe what other “gaps” or aspects of the gap need to be considered.  -Why information on criteria ‘X’ was not available?  -Why criteria ‘X’ or ‘Y’ was not undertaken, considered, or followed? | | **FREE TEXT OPTION (MAX 250 WORDS)**  If the answer was “NO” or “Not possible to assess” or if the question is “Not considered relevant to that specific initiative” to the previous questions please provide additional specific information. Examples include: limited detail in the report, lack of clarity, or ambiguity of the ‘who’, ’what’, ’when’ and ‘how’.  Please also describe what other “gaps” or aspects of the gap need to be considered.  -Why information on criteria ‘X’ was not available?  -Why criteria ‘X’ or ‘Y’ was not undertaken, considered, or followed? | |

**Definitions WP2 used in gap analysis tool**

**The following definitions are provided as a guide to assist in understanding the context that each question is being asked in. Examples given are not prescriptive or exhaustive**

| **Concept** | **Definition** |
| --- | --- |
| Framework/guidance | High level description of key steps, concepts and principles for patient engagement, to be followed by partners. It can consist of several processes (methods), practices or guidances. It can be formal (imposed by constituent bodies) or informal (agreed by a consortium or community group). |
| Processes | A process would likely be based upon existing frameworks or guidances. It describes discrete steps to achieve a certain outcome. |
| Patient engagement case studies | Describes individual experience, of one person, organization, or with one setup/activity, and therefore not automatically qualifies for generalization/use as a general process. It can be a new approach to patient engagement without any link to existing frameworks or guidances. |
| Compensation | Compensation could include reimbursement of expenses for travel, time missed from work, subsistence, child/elderly care, stipends |
| Vulnerable populations | Those considered in PARADIGM as; elderly, people with dementia, young people and their carers/guardian, those with rare diseases, or other underrepresented groups |
| Accessibility | Variables of the patients being engaged with that need to be considered and accounted for. E.g.; physical (impairments), intellectual (impairments), language (appropriate for age, gender, disease and reading level), structural (venue, time), technology (assisted technology, large type, audiovisual, IT) |
| Transparency | The availability of full information on invested resources, procedures, or otherwise required for open collaboration, cooperation, and collective decision making. It is about transparency concerning the PE activity towards the people involved. |
| Methods | Formats or tools used to perform and support the PE activity. E.g.: questionnaires, focus group discussions, interviews, CAPs, etc. |
| Mechanisms | The elements of the PE activity that can be altered to fit the specific needs of patient populations involved. E.g. meetings, workshops, training sessions |
| Tools | The materials used in a PE activity. E.g. roles and responsibility charts, procedural guidance documents, protocols) |
| Lay patients | Persons with personal experience of living with a disease. They may or may not have technical knowledge in R&D or regulatory processes, but their main role is to contribute with their subjective disease and treatment experience. |
| Carers | Persons supporting individual patients such as family members as well as paid or volunteer helpers. |
| Patient advocates | Persons who have the insight and experience in supporting a larger population of patients living with a specific disease. They may or may not be affiliated with an organisation. |
| Patient Organisation Representatives | Persons who are mandated to represent and express the collective views of a patient organisation on a specific issue or disease area. |
| Patient experts or trained patients | In addition to disease-specific expertise, have the technical knowledge in R&D and/or regulatory affairs through training or experience, for example EUPATI Fellows who have been trained by EUPATI on the full spectrum of medicines R&D. |
| Impact/direct outcomes | Direct outcomes of the patient engagement activity, e.g. consensus on the result of the collaborative effort, input and perspectives of all stakeholders are reflected in the results, better implementation of the results, etc. |
| Indirect outcomes | Indirect outcomes for all stakeholders involved  E.g. development of mutual trust, new insights which can improve/guide future PE activities, mutual learning, empowerment, etc. |
| Informing | Patients or patient representatives were only informed in the PE activity. |
| Consulting | Patients or patient representatives were consulted in the PE activity. They have power to influence, but no formal decision power. |
| Deciding | Patient or patient representatives were consulted and had decision making power. |

**Supplementary table 1. Tabulated version of the gap tool used by reviewers of PE initiatives in the gap analysis**

Criteria were mapped under 14 themes. Each criteria had a question and answer structure with multiple choice of answers. Due to the different structure of documents available for in depth review (e.g. framework/guidance, a process/methods document, or a case study) the forty-six assessment questions were structured slightly differently depending on whether the reviewer was assessing material from a i) guidance/framework/process, or ii) case study.

| **Is the initiative an industry or community/patient led one:** | **Response number** |
| --- | --- |
| Industry led initiative | 35 |
| Community/patient led initiative | 10 |
| Co-led by industry and community/patients, please name the initiative | 13 |
| Other | 12 |
| (‘Other’ not specified) | 0 |
| **What phase in the lifecycle of medicines research and development (R&D) is the initiative focused on [multiple answers are possible]?** | |
| Research and priority setting | 31 |
| Design of clinical trials | 49 |
| Early Dialogue with regulators and HTA bodies | 15 |
| It is not possible to assess based on the available information. | 0 |
| Other | 8 |
| (‘Other’ Post-marketing, Dialogue with regulators/HTAs but doesn't qualify as 'early' given the subject matter, interpretation of research results) | |
| **What is the timeline of the initiative?** | |
| Planned, but not yet started | 0 |
| Currently on-going / underway | 18 |
| Completed between and 1 and 3 years ago | 0 |
| Completed < 1 year ago | 10 |
| Completed < 3 years ago | 0 |
| Completed > 1 year ago | 26 |
| It is not possible to assess based on the available information | 2 |
| Completed > 3 years ago, please specify | 14 |
| **In what geography does the initiative cover?** | |
| Global | 25 |
| Regional (please: specify region(s)) | 13 |
| National (please: specify country or countries) | 27 |
| It is not possible to assess based on the available information. | 5 |
| **What is the (medical) scope of the initiative?** | |
| Specific disease area | 25 |
| Therapeutic area specific | 10 |
| More than one therapeutic area | 8 |
| Cross cutting theme across conditions and therapeutic areas | 24 |
| It is not possible to assess based on the available information | 0 |
| Does not apply to this initiative | 3 |
| **Please specify the patient populations involved [multiple answers are possible]:** | |
| Adults | 50 |
| Elderly | 16 |
| Children and Young People | 15 |
| Parent, guardian, or caregiver | 18 |
| It is not possible to assess based on the available information. | 8 |
| Other (please specify) | 4 |
| (‘Other’ Underrepresented (e.g. African Americans), pregnant women(2x), It refers mostly to the involvement of the minorities, community and community leaders, specifically those living in US) | |
| **Please specify the type of patients involved in the initiative [multiple answers are possible]:** | |
| Lay patients | 33 |
| Carers | 22 |
| Patient advocates | 33 |
| Trained patients / patient experts (trained in R&D) | 22 |
| Patient Organisation Representatives | 33 |
| It is not possible to assess based on the available information. | 7 |
| Other (please specify) | 15 |
| (‘Other’ Guidelines for professionals to involve minorities, All types, Health Watch reps (patient access watch dog organization),  Children some with a medical condition, some healthy, Patients. I don't know if they are lay or 'trained').  **Please specify whether this initiative involved a vulnerable population or populations:** | |
| No | 36 |
| Yes | 21 |
| Does not apply to this initiative | 13 |
| **Please specify the level of involvement [multiple answers are possible]:** | |
| Informing | 30 |
| Consulting | 54 |
| Deciding | 14 |
| It is not possible to assess based on the available information. | 3 |
| **Which methods were used or are proposed to be used to involve patients in the initiative [multiple answers are possible]:** | |
| Interviews | 22 |
| Questionnaires/ survey | 18 |
| Consultation | 18 |
| Focus group discussion | 20 |
| Co-creation | 15 |
| Advisory board | 19 |
| It is not possible to assess based on the available information. | 5 |
| Does not apply to this initiative | 0 |
| Other (please specify) | 21 |
| **Do you consider the initiative to be a: [Questions will be slightly different for framework/guidance, process and individual case study]** | |
| Framework/guidance (including additional tools embedded in them) | 18 |
| Processes (tools, SOPs, protocols and templates) | 12 |
| Individual case studies (that describe in part or wholly the PE activity from start to finish) | 40 |

**Supplementary Table 2.** **Reviewer responses to the eleven initiative characteristic questions**

Cumulated responses to seventy initiates that underwent in depth review and analysis. These questions were compulsory and answered at the start of the gap tool before the forty-six assessment questions were answered. Some questions were multiple choice, therefore more than one answer was possible for some questio
